# First checklist of the chrysidid wasps (Hymenoptera, Chrysididae) of Mongolia, with description of new species

**DOI:** 10.3897/zookeys.999.58536

**Published:** 2020-11-30

**Authors:** Paolo Rosa, Maxim Yu. Proshchalykin, Marek Halada, Ulykpan Aibek

**Affiliations:** 1 Via Belvedere 8/d, I–20881 Bernareggio (MB), Italy Unaffiliated Bernareggio Italy; 2 Federal Scientific Centre for East Asian Terrestrial Biodiversity, Far Eastern Branch of Russian Academy of Sciences, Vladivostok 690022, Russia Federal Scientific Centre for East Asian Terrestrial Biodiversity, Far Eastern Branch of Russian Academy of Sciences Vladivostok Russia; 3 Milady Horákové 74 37012 České Budějovice, Czeck Republic Unaffiliated České Budějovice Czech Republic; 4 National University of Mongolia, Ulaanbaatar 210646, Mongolia National University of Mongolia Ulaanbaatar Mongolia

**Keywords:** Catalogue, Central Asia, new records, Palaearctic region

## Abstract

An annotated checklist of the Chrysididae from Mongolia is provided. A revision of the bibliographical data is provied, since most of the collecting localities published for “Mongolia” refer to places currently located in China. The known Mongolian cuckoo wasp fauna counts 90 species in 18 genera and two subfamilies. Four genera and 57 species are recorded for the first time, including two species here described as new for science: *Cleptes mongolicus* Rosa, Halada & Agnoli, **sp. nov.** (Dornod) and *Spinolia spinosa* Rosa & Halada, **sp. nov.** (Bayankhongor).

## Introduction

**Figure 1.**
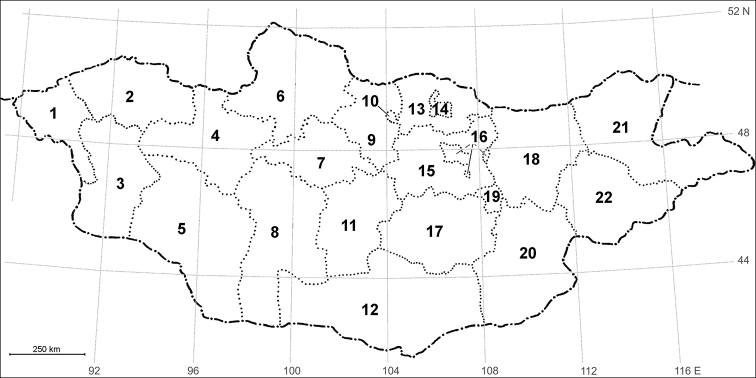
Administrative map of Mongolia (from Dathe and Proshchalykin (2016) and Proshchalykin (2017) modified). Aimags: **1** Bayan-Ulgii **2** Uvs **3** Khovd **4** Zavkhan **5** Govi-Altai **6** Khuvsgul **7** Arkhangai **8** Bayankhongor **9** Bulgan **10** Orkhon **11** Uvurkhangai **12** Umnugovi **13** Selenge **14** Darkhan-Uul **15** Tuv **16** Ulaanbaatar **17** Dundgovi **18** Khentii **19** Govi-Sümber **20** Dornogovi **21** Dornod **22** Sukhbaatar.

Mongolia is a large landlocked country in eastern Central Asia, covering 1,564,100 km². Politically, Mongolia is divided into 21 provinces named “aimags” with the capital Ulaanbaatar (Fig. 1). It is bordered by Russia to the north and China to the south, east, and west. Geographically and climatologically, it is an area of contrasts and extremes, between cold mountainous regions up to 4,000 m a.s.l. to the north and west and one of the largest deserts of the world in the south, the Gobi Desert. Most of the country is located on high plateaus, covered by steppes and extensive forested areas. It has an extreme continental climate with long, cold winters and short hot summers, during which most of its annual precipitation falls (Lavrenko 1979; Dathe and Proshchalykin 2016).

Mongolian cuckoo wasps are scarcely known and a few occasional records are found in the literature (Rosa 2017a). Only one article (Móczár 1967) deals with Mongolian material collected by Dr Z. Kaszab during his entomological excursions in this country (1963–1968). Other scattered findings have been published (du Buysson 1901; Semenov-Tian-Shanskij 1912, 1932, 1967; Semenov-Tian-Shanskij and Nikol’skaya 1954; Linsenmaier 1997a; Rosa et al. 2017a, b), while most of the remaining bibliographical data recorded for “Mongolia” actually refer to localities currently included in China (Inner Mongolia, Xinjiang, Gansu) (du Buysson 1893; Radoszkowski 1877, 1891; Mocsáry 1890; Dalla Torre 1892; Bischoff 1913; Hammer 1936; Tsuneki 1947, 1953a; Linsenmaier 1959, 1968; Semenov-Tian-Shanskij 1967; Kimsey and Bohart 1991; Rosa et al. 2014, 2015). Approximately 30 species were properly recorded from Mongolia so far (Rosa 2017a) and we here add 57 new records for this country, mostly based on the materials collected by Czech entomologists (M. Halada, J. Halada, J. Straka, and M. Kadlecová) in 2003–2007 and mainly housed in the private collections of MH (České Budějovice, Czech Republic) and PR (Bernareggio, Italy). Other new records were found during the examination of the Chrysididae collection housed at the Zoological Institute in St. Petersburg (Russia, ZIN) and based on the material collected during the expeditions of V. Roborovskij and P. Kozlov in 1895 and P. Kozlov in 1926. Finally, a few specimens were examined from the material collected in Mongolia by Soviet-Mongolian expeditions in 1967–1982. Soviet-Mongolian expedition were conducted from 1967 to 1983 and led to the collection of extensive entomological material, which became the basis for the publication of numerous articles and books (including Insects of Mongolia in eleven volumes), devoted to the study of various insects families (Proshchalykin and Kuhlmann 2015), although the Chrysididae were never examined by anyone. Large part of the cuckoo wasps collected during these entomological expeditions is still unprepared and unidentified.

Unpublished distributional records from Mongolia were recently published in the volume on Russian Chrysididae (Rosa et al. 2019), for a better understanding of the distribution of the Asian species, but exact localities were omitted because they were not of interest for that publication. We here report the precise data of species recorded for the first time in Rosa et al. 2019, which are mostly based on material housed in the Linsenmaier collection (Luzern, Switzerland).

In the present paper, based on a comprehensive study of specimens (including primary types) deposited in various collections, we report additional records of 72 species, with two species described as new and 55 species recorded from Mongolia for the first time, resulting in a total number of 90 cuckoo wasps species known from this country (Table 1).

**Table 1.** Records of Mongolian cuckoo wasp species by aimags.

No.	Species	Aimags
1.	*Chrysis aestiva* Dahlbom, 1854	7
2.	*Chrysis angustula* Schenck, 1856	7, 15
3.	*Chrysis asahinai* Tsuneki, 1950	8, 9, 12, 15, 20, 22
4.	*Chrysis belokobylskiji* Rosa, 2019	4, 12, 15
5.	*Chrysis brevitarsis* Thomson, 1870	9
6.	*Chrysis castigata* Linsenmaier, 1959	13, 15
7.	*Chrysis chinensis* Mocsáry, 1912	7, 13, 15
8.	*Chrysis consanguinea* Mocsáry, 1889	4, 7, 9, 13, 15, 16, 18, 21, 22
9.	*Chrysis dauriana* Linsenmaier, 1959	4, 7–9, 13, 18
10.	*Chrysis equestris* Dahlbom, 1854	7, 13
11.	*Chrysis fulgida* Linnaeus, 1761	7, 13, 15
12.	*Chrysis ignita* (Linnaeus, 1758)	9
13.	*Chrysis illecebrosa* Semenov, 1967	12
14.	*Chrysis illigeri* Wesmael, 1839	13, 15
15.	*Chrysis ismaeli* Semenov, 1967	12, 20, 21
16.	*Chrysis jaxartis* Semenov, 1910	12, 13, 15, 18, 21
17.	*Chrysis leptomandibularis* Niehuis, 2000	15
18.	*Chrysis mane* Semenov, 1912	15
19.	*Chrysis matutina* Semenov, 1967	7
20.	*Chrysis mediata* Linsenmaier, 1951	15
21.	*Chrysis mocsaryi* Radoszkowski, 1889	3
22.	*Chrysis mysticalis* Linsenmaier, 1959	4, 7, 9, 15, 20
23.	*Chrysis nox* Semenov, 1954	5, 15
24.	*Chrysis pavesii* Rosa, 2017	5, 15
25.	*Chrysis priapus* Rosa, 2018	5
26.	*Chrysis pseudobrevitarsis* Linsenmaier, 1951	7, 15
27.	*Chrysis pupilla* Semenov, 1967	12
28.	*Chrysis rutilans* Olivier, 1791	15
29.	*Chrysis schencki* Linsenmaier, 1968	7, 9
30.	*Chrysis sibirica* Rosa, 2017	7
31.	*Chrysis solida* Haupt, 1957	21
32.	*Chrysis splendidula unica* Radoszkowski, 1891	7
33.	*Chrysis subcoriacea* Linsenmaier, 1959	7
34.	*Chrysis viridula* Linnaeus, 1761	15
35.	*Chrysura dichroa* (Dahlbom, 1854)	4
36.	*Chrysura ignifrons* (Brullé, 1833)	4
37.	*Cleptes dauriensis* Móczár, 1997	3, 8, 11
38.	*Cleptes mongolicus* Rosa, Halada, & Agnoli, sp. nov.	21
39.	*Colpopyga nesterovi* Rosa, 2017	21
40.	*Elampus albipennis* (Mocsáry, 1889)	7, 20
41.	*Elampus coloratus* Rosa, 2017	22
42.	*Elampus montanus* (Mocsáry, 1890)	20
43.	*Elampus panzeri* (Fabricius, 1804)	4, 7
44.	*Elampus sanzii* Gogorza, 1887	15
45.	*Elampus spinifemoris* (Móczár, 1967)	11
46.	*Euchroeus mongolicus* Tsuneki, 1947	5, 11, 12
47.	*Euchroeus orientis* Semenov, 1910	22
48.	*Hedychridium ardens* (Coquebert, 1801)	4, 7, 8, 11, 13, 16, 18, 21, 22
49.	*Hedychridium asianum* Linsenmaier, 1997	7–9, 16
50.	*Hedychridium belokobylskiji* Rosa, 2017	15
51.	*Hedychridium cupreum* (Dahlbom, 1845)	4, 5, 8, 11, 12, 15, 20
52.	*Hedychridium gabriellae* Rosa, 2017	8, 15, 20
53.	*Hedychridium longigena* Rosa, 2017	8, 9, 13, 15, 18, 20, 21
54.	*Hedychridium propodeale* Rosa, 2017	5
55.	*Hedychridium roseum* (Rossi, 1790)	7, 20–22
56.	*Hedychrum chalybaeum* Dahlbom, 1854	5, 8, 13, 15, 16, 21, 22
57.	*Hedychrum gerstaeckeri* Chevrier, 1869	13, 15, 18
58.	*Hedychrum lama* du Buysson, 1891	3
59.	*Hedychrum longicolle* Abeille de Perrin, 1877	9, 12, 15, 21, 22
60.	*Hedychrum nobile* (Scopoli, 1763)	4, 7, 13, 15
61.	*Hedychrum rutilans ermak* Semenov, 1967	7, 13, 15, 21, 22
62.	*Holopyga generosa asiatica* Trautmann, 1926	13
63.	*Holopyga kaszabi* Móczár, 1967	11, 12, 20
64.	*Holopyga minuma* Linsenmaier, 1959	21, 22
65.	*Omalus aeneus* (Fabricius, 1787)	15, 16
66.	*Omalus berezovskii* (Semenov, 1932)	16
67.	*Omalus margianus* (Semenov, 1932)	7–9, 15, 22
68.	*Omalus miramae* (Semenov, 1932)	8, 20, 22
69.	*Omalus stella* (Semenov, 1932)	7, 11, 15
70.	*Parnopes glasunowi* Semenov, 1901	3
71.	*Parnopes popovii* Eversmann, 1858	7, 9, 12, 15, 20–22
72.	*Pentachrysis amoena* (Eversmann, 1858)	without locality
73.	*Philoctetes bogdanovii* (Radoszkowski, 1877)	7
74.	*Philoctetes cynthiae* Rosa, 2017	8, 11, 16, 22
75.	*Philoctetes diakonovi* (Semenov, 1932)	20
76.	*Philoctetes lyubae* Rosa, 2017	20
77.	*Philoctetes mongolicus* (du Buysson, 1901)	7, 8, 11, 15, 16, 18, 22
78.	*Philoctetes shokalskii* (Semenov, 1932)	8, 11, 12, 15, 16, 18–22
79.	*Pseudochrysis gengiskhan* Rosa, 2017	8, 9, 13, 15, 21, 22
80.	*Pseudochrysis neglecta* (Shuckard, 1837)	15
81.	*Pseudomalus auratus nigridorsus* (Tsuneki, 1953)	4, 9, 15, 18
82.	*Pseudomalus corensis* (Uchida, 1927)	9, 13, 15, 16, 18, 21
83.	*Pseudomalus punctatus* (Uchida, 1927)	9, 15, 18, 21
84.	*Pseudomalus pusillus* (Fabricius, 1804)	8, 9, 11–13, 15, 18, 21
85.	*Spinolia spinosa* Rosa & Halada, sp. nov.	8
86.	*Spinolia unicolor* (Dahlbom, 1831)	5
87.	*Stilbum calens* (Fabricius, 1781)	7, 9, 11, 15, 20
88.	*Trichrysis cyanea* (Linnaeus, 1758)	8, 13, 15
89.	*Trichrysis pellucida* (du Buysson, 1887)	without locality
90.	*Trichrysis secernenda* (Mocsáry, 1912)	13

Comment. Aimag designation as in Fig. 1.

## Materials and methods

Terminology follows Lanes et al. (2020), Hymenoptera Anatomy Ontology (HAO 2020), and partly Kimsey and Bohart (1991). Abbreviations used in the descriptions are as follows:

**F1, F2, F3, etc.** flagellomeres 1, 2, 3, etc., respectively;

**l/w** length/width;

**MOD** anterior ocellus diameter;

**MS** malar space, the shortest distance between base of mandible and lower margin of compound eye;

**OOL** the shortest distance between posterior ocellus and compound eye;

**P** pedicel;

**PD** puncture diameter;

**POL** the shortest distance between posterior ocelli;

**T1–T5** metasomal terga numbered consecutively, starting with 1 at the second abdominal segment.

Pictures of the types were taken with Nikon D700 connected to the microscope Togal SCZ and stacked with the software Combine ZP.

The checklist follows the genera subdivision proposed by Kimsey and Bohart (1991), with few exceptions for some genera (e.g., *Euchroeus* Latreille, 1809, *Pseudochrysis* Semenov, 1891 and *Colpopyga* Semenov, 1954). The species are listed alphabetically. We have used the following abbreviations for collectors: **JH** – J. Halada; **JS** – J. Straka; **MH** – M. Halada; **MK** – M. Kadlecová. An asterisk (*) marks the new records.

Types and other specimens are deposited in the following Institutions and private collections:

**EIHU**Entomology Institute, Hokkaido University (Japan);

**HNHM**Hungarian Natural History Museum, Zoological Department, Budapest (Hungary);

**ISEA-PAS** Institute of Systematics and Evolution of Animals, Polish Academy of Sciences, Kraków (Poland);

**LSL** Linnean Society, London (England);

**MCNM** Museo National de Ciencias Naturales, Madrid (Spain);

**MFN** Museum für Naturkunde, Berlin (Germany);

**MHNG**Museum d’Histoire Naturelle, Geneva (Switzerland);

**MNHN**National Museum of Natural History, Paris (France);

**MSNG**Museo di Storia Naturale, Genova (Italy);

**NHMUK**British Museum of Natural History, London (UK);

**NHMW**Museum of Natural History, Vienna (Austria);

**NIAS** National Institute of Agro-Environmetal Science, Tsukuba (Japan);

**NMLS**Natur Museum, Luzern (Switzerland);

**OMNH**Osaka Museum of Natural History, Osaka (Japan);

**ZIN**Zoological Institute, Russian Academy of Sciences, St. Petersburg (Russia);

**ZMMU**Zoological Museum of Moscow Lomonosov State University (Russia);

**ZMUL**Zoologiska Museet, Lund Zoological Museum, University of Lund (Sweden);

**GLAC** G.L. Agnoli collection (Bologna, Italy);

**MHC** M. Halada collection (České Budějovice, Czech Republic);

**PRC** P. Rosa collection (Bernareggio, Italy);

**UKC** U. Koschwitz collection (Eppenbraun, Germany).

## Results

### Taxa from Mongolia

#### Subfamily Cleptinae

##### Genus *Cleptes* Latreille, 1802

*Cleptes* Latreille, 1802: 316. Type species: *Sphex semiaurata* Linnaeus, 1761 [= *Cleptes semiauratus* (Linnaeus, 1761)], by monotypy.

###### Cleptes dauriensis

Taxon classificationAnimaliaHymenopteraChrysididae

Móczár, 1997

0FA19F1D-FEBD-5567-A083-9689B8EEB63C

Cleptes (Cleptes) dauriensis Móczár, 1997: 36. Holotype ♀: Russia: Dauria, leg. F. Sahlb., “*Cleptes* n. sp. *nitidulo* Fbr. aff.”, Holotype Cleptes dauriensis ♀ Móczár n. sp. det. Móczár 1995” (Hym. Typ. No. 3845 Mus. Budapest) (HMNH).
Cleptes dauriensis : Rosa 2017a: 288. Rosa et al. 2019: 310 (Mongolia, Figs 4, 5).

####### Material examined.

Mongolia: *Khovd*, 1 ♂, Bodongin-Gol River, 12 km SW Altai, 22.VII.1970, leg. M. Kozlov (ZIN); *Uvurkhangai*, 1 ♀, 12 km E of Arvaykheer, 46°22'N, 102°49'E, 1800 m, 3.VII.2004, leg. JH (GLAC); *Bayankhongor*, 1 ♂, 16 km SW of Bayankhongor, 46°13'N, 100°30'E, 2165 m, 10.VII.2004, leg. JH (GLAC).

####### Distribution.

Mongolia (Bayankhongor, Khovd, Uvurkhangai); Russia (Zabaikalskii Terr.) (Rosa 2017a).

###### Cleptes mongolicus

Taxon classificationAnimaliaHymenopteraChrysididae

Rosa, Halada & Agnoli sp. nov.

DAB361FF-A06A-5BAC-A41B-DC0DFA0A4D9D

http://zoobank.org/73389B93-F683-41CC-84AC-3E16ED9B3000

Figures 2 , 3

####### Type material.

***Holotype***: ♀, Mongolia: *Dornod*, 100 km W of Choibalsan, 820 m, 23.VII.2007, leg. M. Halada (ZIN). ***Paratypes***: 1 ♂, same collecting locality and date (GLAC); 1 ♂, 20 km W of Choibalsan, 48°01'N, 114°14'E, 800 m, 24.VII.2007, leg. M. Halada (PRC).

####### Diagnosis.

*Cleptes mongolicus* sp. nov. belongs to the *C. nitidulus* species group, based on the pronotum without posterior pit row and without longitudinal median sulcus or posterior median keel. It is closely related only to *C. margaritae* Móczár, 2000 from Tajikistan, for its general habitus and colouration. The latter belongs to the *C. satoi* group (Móczár 2000), for the modified pronotal structure, without posterior transversal groove, but with a posteromedian longitudinal keel. Besides the unmodified pronotum, the female of *C. mongolicus* sp. nov. can be easily separated from the female of *C. margaritae* by: a) pubescence whitish, shorter on metasoma (max 2.5 MOD) (vs. blackish, longer on metasoma, up to 3 MOD); b) punctation on metasoma with polished T1, shallow and sparse tiny punctures on T2, double punctures on T3 (vs. scattered punctate on T1, densely and evenly punctate on T2 and T3); c) colouration: head entirely black; propodeum entirely blue; T3 and T4 laterally blue; pedicel and F1 yellow; femora apically, tibiae and tarsi yellow (vs. head blue; propodeum black with median blue spot; T3 and T4 fully black; pedicel and flagellum dark brown). The male of *Cl. margaritae* is currently unknown.

####### Description.

**Figure 2.**
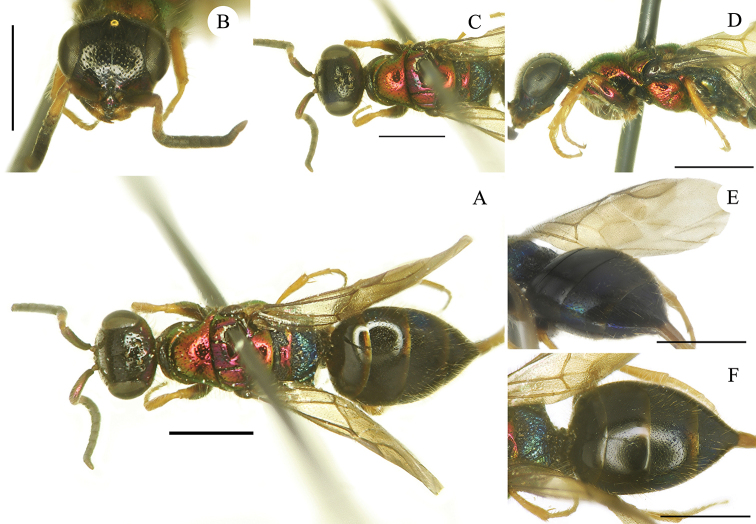
*Cleptes mongolicus* sp. nov., female, holotype **A** habitus, dorsal view **B** head, frontal view **C** head and mesosoma, dorsal view **D** head and mesosoma, lateral view **E** metasoma, postero-lateral view **F** metasoma, dorso-lateral view. Scale bars: 1.0 mm.

**Female.** Holotype (Fig. 2A–F). Body length 4.6 mm. Forewing length 2.7 mm. POL = 2.2 MOD; OOL = 2.7 MOD. MS = 2.0 MOD. P:F1:F2:F3 = 1.0:1.0:0.7:0.7. F1 1.5 × as long as wide, F2 1.1 × as long as wide. ***Head*.** Head in frontal view 1.2 × as broad as long between lower edge of clypeus and vertex. Face and vertex with small, even, and sparse punctures (1–4 PD) (Fig. 2B). Clypeal lower margin simple, unmodified, 2 MOD width, without acute teeth at corners; lateral edges subparallel. Frontal sulcus broad and deep in the first part, from anterior ocellus to mid of face, faint in the second half, from mid-face to the clypeal margin (Fig. 2B). Mandibles tridentate. Ocellar triangle isosceles, without post-ocellar sulcus. Postero-lateral pits close to posterior ocelli deep and elongate. Pedicel as long as F1. Malar spaces elongate (2.0 MOD). ***Mesosoma*.** Pronotum unmodified; pronotal neck finely striated transversally; posterior margin of pronotum simple, without transverse row of pits or median keel. Pronotum with small punctures similar to those on vertex. Mesoscutum and mesoscutellum scarcely punctate, with tiny and scattered punctures (Fig. 2C), largely impunctate; notauli and parapsidal lines deep and complete. Mesopleuron with small, deep punctures; transversely aligned medially; with short, deep scrobal sulcus on posterior half (Fig. 2D). Metascutellum noticeably reduced by large metanotal trough and by deep and large anteromedian suture. Metapleuron transversely striate. Metapostnotum (dorsal surface of metapectal-propodeal complex) short, irregularly reticulate, with large foveae along posterior margin, before the propodeal declivity. Propodeal posterior projections short, stout, and divergent. Wing veins and cells unmodified. ***Metasoma*.** All metasomal terga with impunctate, brownish stripe along posterior margin (Fig. 2F); T1 mostly impunctate, with a few, sparse, tiny punctures; T2 with even, sparse, small punctures (3–5 PD), posteromedially polished; T3 with dense, irregular and double punctation; scattered to polished toward the apical margin; T4 with large, scattered punctures. ***Colouration*.** Head black, with violet reflections medially on clypeus; scapus dorsally violet, ventrally brownish without metallic reflections; P light brown and F1 yellow; other flagellomeres dark brown to blackish. Mandible dark brown, medially yellowish. Pronotal neck medially black; pronotum, mesonotum, mesopleuron, metanotum (excluding black anterior suture and axillary trough), metapleuron metallic red with purple reflections dorsally; propodeum dorsally blue, propodeal declivity black; body ventrally black. Metasoma entirely black; apical margin of each tergum with brownish stripe; laterally on T3 with feeble green reflections; laterally on T4 with extended blue reflections (Fig. 2E). Tegulae brown. Legs with tibiae and tarsi yellowish; coxae red to golden; profemur anteriorly metallic red excluding distal joint; metafemur posteriorly metallic; other parts brown.

**Figure 3.**
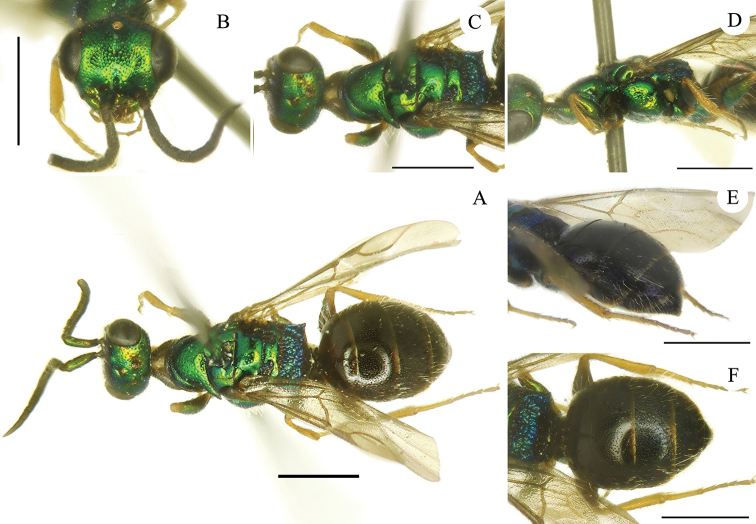
*Cleptes mongolicus* sp. nov., male, paratype **A** habitus, dorsal view **B** head, frontal view **C** head and mesosoma, dorsal view **D** mesosoma, lateral view **E** metasoma, postero-lateral view **F** metasoma, dorsal view. Scale bars: 1.0 mm.

**Male.** Paratypes. Body length 4.0–4.2 mm. POL = 1.6 MOD; OOL = 1.0 MOD. MS = 1.9 MOD. P:F1:F2:F3 = 1.0:1.4:0.9:0.9. F1 3.5 × as long as wide (width taken at distal apex), F2 1.5×. ***Head*.** Head in frontal view 1.3 × as broad as long between lower edge of clypeus and vertex. Face and vertex with small, even, and denser punctures (1–2 PD) compared to female (Fig. 3B). Frontal sulcus narrow and visible in the first part, from anterior ocellus to brow, faint in the second half, from mid-face to the clypeal margin (Fig. 3B). Lower face medially with punctures more spaced 4–5 PD. Ocellar triangle, post-ocellar sulcus, and posterolateral pits similar to female. F1 1.5 × as long as P. ***Mesosoma*.** Punctation overall similar to that of female; metascutellum larger, with narrow anteromedian mesoscutellar-metascutal suture; metapleuron polished. Other characters as in female. ***Metasoma*.** T1 with denser (2–5 PD), tiny punctures; T2 with even, denser (1–3 PD), small punctures (3–5 PD), posteromedially sparser to polished; T3 with dense, irregular and double punctation; scattered to polished toward the apical margin; T4 with similar punctures; T5 almost polished, with scattered punctures. ***Colouration*.** Species sexually dimorphic with head and mesosoma bright green, including ventral side; propodeum blue. Mandible metallic green from base to half length. Scapus green, pedicel and flagellum black. Metasoma entirely black, with terga apically brownish and laterally with feeble blue reflections on T3 and T4 (Fig. 3E). Tegulae brown. Coxae and femora medially green; trochanters brown, femora distally and tarsi yellowish.

####### Etymology.

The specific epithet is named after the country of origin.

####### Distribution.

Mongolia (Dornod).

#### Subfamily Chrysidinae

**Tribe Chrysidini**

##### Genus *Chrysis* Linnaeus, 1761

*Chrysis* Linnaeus, 1761: 414. Type species: *Sphex ignita* Linnaeus, 1758 [= *Chrysis ignita* (Linnaeus, 1758)], by subsequent designation of Latreille 1810: 437.

*Tetrachrysis* Lichtenstein, 1876: 27. Type species: *Chrysis aeruginosa* Dahlbom, 1854, by subsequent designation of Ashmead 1902: 226. Synonymized by Linsenmaier 1959: 91.

###### Chrysis aestiva

Taxon classificationAnimaliaHymenopteraChrysididae

Dahlbom, 1854

AEE4D3F6-6EF6-571E-83D9-3949A5A4B9E4

Chrysis aestiva Dahlbom, 1854: 286. Holotype ♀; Greece: Rhodes (Berlin ?) (aestiva group).

####### Material examined.

Mongolia: *Arkhangai*, 1 ♂, 90 km NE of Tsetserleg, 48°03'N, 102°25'E, 27.VII.2005, leg. JH (MHC).

####### Distribution.

*Mongolia (Arkhangai); Asiatic-European, from Caucasus, Turkey, Greece, Iran, Palestine, European part of Russia to Mongolia (Rosa et al. 2019, present record).

####### Remarks.

This is the most eastern record for *Chrysis aestiva*.

###### Chrysis angustula

Taxon classificationAnimaliaHymenopteraChrysididae

Schenck, 1856

223D1189-8F3B-5019-81C2-120F40534B84

Chrysis angustula Schenck, 1856: 28. Lectotype ♀ (designated by Morgan 1984: 9); Germany: former Duchy of Nassau (Frankfurt) (ignita group).

####### Material examined.

Mongolia: *Arkhangai*, 5 ♀♀, 1 ♂, Chuluut Gol River, 47°48'N, 100°19'E, 23.VII.2005, leg. JH (MHC); 4 ♀♀, 1 ♂, 70 km NE of Tsetserleg, 25.VII.2005, leg. JH (MHC); *Tuv*, 1 ♀, 2 ♂♂, 50 km E of Ulaanbaatar, Tuul River, 22.VI.2003, leg. JH (MHC).

####### Distribution.

*Mongolia (Arkhangai, Tuv); Asiatic-European, from western Europe to China and Russia (Rosa et al. 2019).

###### Chrysis asahinai

Taxon classificationAnimaliaHymenopteraChrysididae

Tsuneki, 1950

66561CA1-3312-586D-A518-10BDD2E4C95C

Chrysis (Tetrachrysis) asahinai Tsuneki, 1950: 80. Holotype ♀; China, Manchuria, 22.VIII.1938, leg. S. Asahina (OMNH) (*pulchella* group).
Chrysis asahinai : Móczár 1967: 189 (cat., Mongolia: 1 ♀, Estgobi aimag: Cagan Elis, 800 m, 30 km ESE von Zuun-Bajan, Exp. Dr. Z. Kasab, 1963, nr. 22, 23.VI.1963).

####### Material examined.

Mongolia: *Bayankhongor*, 12 ♂♂, 130 km S of Bayankhongor, 45°03'N, 100°59'E, 1240 m, 6.VII.2004, leg. JH, MK (MHC, PRC); 1 ♀, ibid, Orog Nuur, 6–7.VII.2004, on *Saxaul*, leg. JS (PRC); *Bulgan*, 13 ♀♀, 4 ♂♂, Mongol Els Nat. Res., dunes, 47°24'N, 103°39'E, 31.VII.2005, leg. JH (MHC); *Sukhbaatar*, 1 ♂, 100 km SSW of Baruun-Urt, 1100 m, 30.VII.2007, leg. MH (MHC); *Tuv*, 2 ♀♀, 2 ♂♂, 75 km W of Ulaanbaatar, dunes, 2.VIII.2005, leg. JH (MHC); 39 ♀♀, 37 ♂♂, 70 km W of Ulaanbaatar, 1070 m, dunes, 16.VIII.2007, leg. JH, MH (MHC); *Umnugovi*, 1 ♂, Gobi, 100 km SW of Dalanzadgad, Bayanzag, on *Saxaul*, 1–2.VII.2003, leg. JH (MHC).

####### Distribution.

Mongolia (*Bayankhongor, *Bulgan, Dornogovi, *Sukhbaatar, *Tuv, *Umnugovi); China (Liaoning) (Rosa et al. 2014).

###### Chrysis belokobylskiji

Taxon classificationAnimaliaHymenopteraChrysididae

Rosa, 2019

3891D06F-DD86-5B4F-AEF5-4D96F7222DFB

Chrysis belokobylskiji Rosa, 2019: 2. Holotype ♀; Kyrgyzstan: Naryn River near Karakolka (ZIN) (examined); paratypes: 2 ♀♀, 1 ♂ [Mongolia: Nogon-kub, N. Gobi; 50 km E of Ulaanbaatar, Tuul River; 40 km SW of Uliastay]) (*pulchella* group).

####### Material examined.

Mongolia: *Umnugovi*, 1 ♀, Nogon-kub, N. Gobi, 1.VIII.1926, P. Kozlov (ZIN); *Tuv*, 1 ♂, 50 km E of Ulaanbaatar, Tuul River, 22.VI.2003, leg. JH (MHC); *Zavkhan*, 1 ♀, 40 km SW of Uliastay, dunes, 18.VII.2005, leg. JH (MHC).

####### Distribution.

Mongolia (Tuv, Umnugovi, Zavkhan); China (Qinghai), Kyrgyzstan, Tajikistan (Rosa 2019).

###### Chrysis brevitarsis

Taxon classificationAnimaliaHymenopteraChrysididae

Thomson, 1870

7562EB77-12BF-5469-A482-AB7ED095ADD6

Chrysis brevitarsis Thomson, 1870: 107. Holotype ♀; Sweden: Nerike [= Närke] (Lund) (examined) (ignita group).

####### Material examined.

Mongolia: *Bulgan*, 1 ♀, 137 km NE of Aravaykheer, 47°20'N, 103°40.5'E, 1250 m, 26.VII.2004, leg. JH (MHC).

####### Distribution.

*Mongolia (Bulgan); Asiatic-European, from western Europe to Russia (Rosa et al. 2019).

###### Chrysis castigata

Taxon classificationAnimaliaHymenopteraChrysididae

Linsenmaier, 1959

CF45C6EF-C88A-5FD3-802A-04D45CF4DBEA

Chrysis (Chrysis) exsulans var. asiatica Linsenmaier, 1951: 82. Holotype ♀; Uzbekistan: Ferghana (Budapest) (examined) (ignita group), nom. praeocc., nec Radoszkwoski 1889.
Chrysis (Chrysis) exsulans var. castigata Linsenmaier, 1959: 155. Replacement name for C. asiatica Linsenmaier, 1951.

####### Material examined.

Mongolia: *Selenge*, 2 ♂♂, 90 km N of Ulaanbaatar, Segnez River, 1450 m, 6–8.VII.2003, leg. JH (MHC); *Tuv*, 1 ♂, 50 km N of Ulaanbaatar, E of Mandal, 1180 m, 8–13.VIII.2007, leg. MH (MHC).

####### Distribution.

*Mongolia (Selenge, Tuv); Kazakhstan, Kyrgyzstan, Turkmenistan, Uzbekistan, Russia (Eastern Siberia) (Rosa et al. 2019).

###### Chrysis chinensis

Taxon classificationAnimaliaHymenopteraChrysididae

Mocsáry, 1912

81563555-1A1A-5F3B-93BA-469182A7B1C4

Chrysis (Tetrachrysis) ignita var. chinensis Mocsáry, 1912: 589. Holotype ♀; China: Shanghai (HNHM) (examined) (ignita group).
Chrysis chinensis : Rosa et al. 2019: 109 (cat., Mongolia, without locality, see Material examined).

####### Material examined.

Mongolia: *Arkhangai*, 24 ♂♂, Chuluut Gol River, 47°48'N, 100°19'E, 23.VII.2005, leg. JH (MHC); 2 ♀♀, 4 ♂♂, 90 km NE of Tsetserleg, 48°03'N, 102°25'E, 24.VII.2004, leg. JH (MHC); 2 ♀♀, ibid, 27.VII.2005, leg. JH (MHC); *Tuv*, 1 ♀, 1 ♂, Ulaanbaatar Bog Duul, 11.VII.1983, leg. Karl Bleyl (NMLS); 7 ♀♀, 28 ♂♂, 50 km E of Ulaanbaatar, Tuul River, 22.VI.2003, leg. JH (MHC); 6 ♂♂, Khangayn Mts, 5 km N of Khunt, 20.VII.2005, leg. JH (MHC); *Selenge*, 11 ♀♀, 19 ♂♂, 90 km N of Ulaanbaatar, Segnez River, 1450 m, 6–8.VII.2003, leg. JH (MHC).

####### Distribution.

Mongolia (*Arkhangai, *Selenge, *Tuv); Asiatic-European, from western Europe (Switzerland) to China (Helongjiang, Shanghai) (Linsenmaier 1959).

####### Remarks.

This species was previously reported from Mongolia (Rosa et al. 2019) without exact locality.

###### Chrysis consanguinea

Taxon classificationAnimaliaHymenopteraChrysididae

Mocsáry, 1889

BDDA689D-AC3F-5D71-BF0C-2E5790DA4889

Chrysis (Gonochrysis) consanguinea Mocsáry, 1889: 299. Syntypes ♀♀; Italy: Sicily; Algeria (MHNG) (examined) (viridula group).

####### Material examined.

Mongolia: *Arkhangai*, 1 ♂, Chuluut Gol River, 47°48'N, 100°19'E, 23.VII.2005, leg. JH (MHC); 3 ♀♀, 90 km NE of Tsetserleg, 48°03'N, 102°25'E, 27.VII.2005, leg. JH (MHC); *Bulgan*, 1 ♀, 137 km NE of Aravaykheer, 47°20'N, 103°40.5'E, 1250 m, 2.VII.2004, leg. JH (MHC); *Dornod*, 1 ♀, 100 km W of Choilbalsan, 820 m, 23.VII.2007, leg. JH (MHC); *Khentii*, 4 ♂♂, 100 km NE of Ondorkhaan, Kerulen River, 970 m, 22.VII.2007, leg. MH (MHC); *Selenge*, 1 ♀, 2 ♂♂, 90 km N of Ulaanbaatar, Segnez River, 1450 m, 6–8.VII.2003, leg. JH (MHC); *Sukhbaatar*, 2 ♂♂, 100 km SSW of Baruun-Urt, 1100 m, 30.VII.2007, leg. MH (MHC); *Tuv*, 7 ♂♂, 50 km E of Ulaanbaatar, Tuul River, 22.VI.2003, leg. JH (MHC); 1 ♂, Khangaun Mts, 5 km N of Khunt, 20.VII.2005, leg. JH (MHC); 1 ♀, 50 km N of Ulaanbaatar, E of Mandal, 1180 m, 8–13.VIII.2007, leg. JH (MHC); *Ulaanbaatar*, 1 ♀, 7 km E of Ulaanbaatar, Gachuurt, 47°55'N, 107°06'E, 31.VII.2002, leg. JS (MHC); *Zavkhan*, 1 ♂, 40 km SW of Uliastay, dunes, 18.VII.2005, leg. JH (MHC).

####### Distribution.

*Mongolia (Arkhangai, Bulgan, Dornod, Khentii, Selenge, Sukhbaatar, Tuv, Ulaanbaatar, Zavkhan); Palaearctic, from southern Europe and northern Africa to Eastern Siberia ang Mongolia (Rosa et al. 2019, present records).

###### Chrysis dauriana

Taxon classificationAnimaliaHymenopteraChrysididae

Linsenmaier, 1959

137B485D-37FC-5FA2-BCB4-38B6933A8E8B

Chrysis (Chrysis) cavaleriei ssp. dauriana Linsenmaier, 1959: 112. Holotype ♀; Russia: Dauria (NMLS) (examined) (succincta group). Elevated to species rank by Rosa et al. 2017a: 40.
Chrysis (Tetrachrysis) mongolica Semenov-Tian-Shanskij, 1967: 178, nec Mocsáry, 1914. Holotype ♀; Russia [not Mongolia]; Transbaikalia: Ingoda River (St. Petersburg) (examined). Rosa et al. 2017a: 39 (cat., type series), 155 (Plate 91). Synonymised by Rosa et al. 2017a: 40.
Chrysis mongoliana Bohart in Kimsey and Bohart 1991: 440. Replacement name for Chrysis mongolica Semenov-Tian-Shanskij, 1967: 178, nec Mocsáry 1914.

####### Material examined.

Mongolia: *Arkhangai*, 1 ♀, 90 km NE of Tsetserleg, 48°03'N, 102°25'E, 27.VII.2005, leg. JH (MHC); *Bayankhongor*, 2 ♂♂, 163 km S of Bayankhongor, 46°13'N, 100°30'E, 2165 m, 10.VII.2004, leg. JH (MHC); *Bulgan*, 1 ♂, 170 km W of Ulaanbaatar, dunes, 1070 m, 16.VIII.2007, leg. MH (MHC); *Khentii*, 1 ♀, 100 km NE of Ondorkhaan, Kerulen River, 970 m, 22.VII.2007, leg. MH (MHC); *Selenge*, 4 ♀♀, 2 ♂♂, 90 km N of Ulaanbaatar, Segnez River, 1450 m, 6–8.VII.2003, leg. JH (MHC); *Tuv*, 2 ♀♀, 4 ♂♂, 50 km E of Ulaanbaatar, Tuul River, 22.VI.2003, leg. JH (MHC); 2 ♂♂, ibid, 12.VII.2003, leg. JH (MHC); 10 ♀♀, 6 ♂♂, 50 km N of Ulaanbaatar, E of Mandal, 1180 m, 8–13.VIII.2007, leg. MH (MHC, PRC); *Zavkhan*, 2 ♂♂, 40 km SW of Uliastay, dunes, 18.VII.2005, leg. JH (MHC).

####### Distribution.

Mongolia (*Arkhangai, *Bayankhongor, *Bulgan, *Khentii, *Selenge, *Zavkhan); Russia (Eastern Siberia) (Rosa et al. 2017a).

####### Remarks.

This species was previously reported from Mongolia (Rosa et al. 2019) without exact locality.

###### Chrysis equestris

Taxon classificationAnimaliaHymenopteraChrysididae

Dahlbom, 1854

6DBC0BFA-F658-5F25-AC09-CA485AA6C786

Chrysis equestris Dahlbom, 1854: 307. Holotype ♀; locality unknown [most likely Sweden] (Stockholm) (examined) (*smaragdula* group).

####### Material examined.

Mongolia: *Arkhangai*, 1 ♀, 2 ♂♂, 90 km NE of Tsetserleg, 48°03'N, 102°25'E, 24.VII.2004, leg. JH (MHC); 1 ♀, 1 ♂, ibid, leg. MK (MHC); 1 ♀, 1 ♂, 70 km NE Tsetserleg, 25.VII.2005, leg. JH (MHC); *Selenge*, 1 ♂, 90 km N of Ulaanbaatar, Segnez River, 1450 m, 6–8.VII.2003, leg. JH (MHC).

####### Distribution.

*Mongolia (Arkhangai, Selenge); Asiatic-European, from western Europe to Russia (Rosa et al. 2019).

###### Chrysis fulgida

Taxon classificationAnimaliaHymenopteraChrysididae

Linnaeus, 1761

01844BC8-DB38-5BA0-B72A-5A25C8E21806

Chrysis fulgida Linnaeus, 1761: 415. Lectotype ♀ (designated by Morgan 1984: 9); Sweden: Uppsala (LSL) (ignita group).

####### Material examined.

Mongolia: (*Form A*): *Arkhangai*, 2 ♀♀, 1 ♂, 70 km NE of Tsetserleg, 25.VII.2005, leg. JH (MHC); *Selenge*, 1 ♂, 90 km N of Ulaanbaatar, Segnez River, 1450 m, 6–8.VII.2003, leg. JH (MHC); *Tuv*, 5 ♀♀, 3 ♂♂, 50 km E of Ulaanbaatar, Tuul River, 22.VI.2003, leg. JH (MHC); (*Form B*): *Arkhangai*, 2 ♀♀, 1 ♂, 70 km NE of Tsetserleg, 25.VII.2005, leg. JH (MHC); 1 ♀, 1 ♂, Chuluut Gol River, 47°48'N, 100°19'E, 23.VII.2005, leg. JH (MHC); *Tuv*, 1 ♀, 50 km E of Ulaanbaatar, Tuul River, 22.VI.2003, leg. JH (MHC).

####### Distribution.

*Mongolia (Arkhangai, Selenge, Tuv); Asiatic-European, from Europe to eastern Siberia, Russian Far East and North-East China (Manchuria) (Rosa et al. 2014, 2019).

####### Remarks.

**Figure 4.**
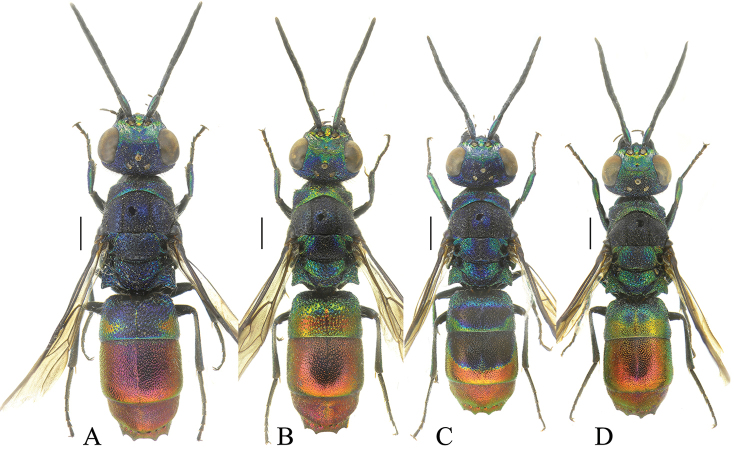
*Chrysis fulgida* Linnaeus, habitus dorsal view **A** form A, ♀ **B** form B, ♀ **C** form A, ♂ **D** form B, ♂. Scale bars: 1.0 mm.

Two distinct colour forms (Fig. 4) are recorded from Mongolia, Siberia and Primorsky Territory (Russia), and Heilongjiang (China). Form A is matching with the typical European *Chrysis fulgida* (Fig. 4A, C). Form B is chromatic different without the typical blue colouration on male and female metasoma and with non-metallic black areas on head vertex and mesosoma (Fig. 4B, D). Male T1 golden-greenish, with or without a narrow transversal green or bluish stripe or patch; T2 red, with or without a basal, narrow black stripe; female T1 golden-greenish, with green to bluish colour on T1 frontal declivity to petiolar insertion. This colour variation has also been observed in specimens from Russia (Siberia and Primorsky Territory) and China (Heilongjiang). The Chinese form was mentioned by Linsenmaier (1968) as *Chrysis aequicolor* Linsenmaier, 1968, which is anyway an unnecessary replacement name for Chrysis fulgida var. concolor Mocsáry, 1912 nec Mocsáry, 1892 (actually male and female of the same taxon). Other evident different morphological characteristics are not recognizable. However, these two forms may represent two sister species, genetically separate, but difficult to identify on the basis of morphological characteristics, as in other known cases of *Chrysis* of the *ignita* group (Paukkunen et al. 2015; Orlovskytė et al. 2016).

###### Chrysis ignita

Taxon classificationAnimaliaHymenopteraChrysididae

(Linnaeus, 1758)

CA3F82A1-F484-5363-83F7-3340C4A7BD14

Sphex ignita Linnaeus, 1758: 571. Lectotype ♀ (designated by Richards 1935); Europe (LSL) (ignita group).
Chrysis ignita : Buyanjargal and Abasheev 2015: 31 (biol. host of Euodynerus dantici, central Mongolia: Khugnu-Khaan Mts, Khugnu-Tarna N.P.).

####### Material examined.

None examined.

####### Remarks.

The identification of *Chrysis ignita* by Buyanjargal and Abasheev (2015) is doubtful and very likely represent another species of the *C. ignita* group or even a member of another species group (e.g., *succincta* group). In fact, the host association with *Euodynerus dantici*, as observed by the two authors, is unusual. *Euodynerus dantici* is known as a possible host for members of the *C. succincta* group (*C. germari* and *C. tristicula* (sub *C. succincta succinctula*) Pauli et al. 2019, supplementary file 4). For example, *C. dauriana* Linsenmaier was erroneously identified as *C. ignita* by several authors, including Trautmann (identification label pinned with the type of *C. dauriana*).

####### Distribution.

Mongolia (Bulgan) [doubtful]; West-Palaearctic: from West Europe to central Asia (Linsenmaier 1997b).

###### Chrysis illecebrosa

Taxon classificationAnimaliaHymenopteraChrysididae

Semenov, 1967

A728A8EB-FE41-5160-8B37-087E90107832

Chrysis (Tetrachrysis) illecebrosa Semenov-Tian-Shanskij, 1967: 166. Holotype ♂; Bugas near Khami, SE from Tian Shan [China, Xinjiang] (ZIN) (examined) (*maculicornis* group).

####### Material examined.

Mongolia: *Umnugovi*, 1 ♂, Deemgin-gobi, 25 km SSO of Khajlastyn-Khuduka, 20.VI.1971, leg. M. Kozlov (ZIN).

####### Distribution.

*Mongolia (Umnugovi); China (Xinjiang) (Rosa et al. 2014).

###### Chrysis illigeri

Taxon classificationAnimaliaHymenopteraChrysididae

Wesmael, 1839

4532A2FC-4222-5E81-9A47-5C0C2D2690C0

Chrysis illigeri Wesmael, 1839: 176. Syntypes ♂♀; Belgium (Bruxelles, MSNG) (examined) (succincta group).

####### Material examined.

Mongolia: *Selenge*, 1 ♂, 90 km N of Ulaanbaatar, Segnez River, 1450 m, 6–8.VII.2003, leg. JH (MHC); *Tuv*, 1 ♀, 50 km N of Ulaanbaatar, E of Mandal, 1180 m, 8–13.VIII.2007, leg. MH (MHC).

####### Distribution.

*Mongolia (Selenge, Tuv); Asiatic-European, from western Europe to Mongolia (present record).

###### Chrysis ismaeli

Taxon classificationAnimaliaHymenopteraChrysididae

Semenov, 1967

439A7EA6-B583-5B71-9ACA-E1D1E3A57B6F

Chrysis (Allochrysis) ismaeli Semenov-Tian-Shanskij, 1967: 124. Holotype ♀; Kazakhstan: Balamurun, Karatau Mountain ridge foothills, leg. V. Kozhantschikov (ZIN) (ear group).

####### Material examined.

Mongolia: *Dornod*, 1 ♂, 100 km W of Choilbalsan, 820 m, 23.VII.2007, leg. MK (MHC); *Dornogovi*, 5 ♂♂, 65 km SE of Chatan-Bulag, 1020 m, 2.VIII.2007, leg. MH (PRC/MHC); *Umnugovi*, 1 ♀, Gobi, Dalanzadgad, 24–26.VI.2003, leg. JH (MHC); 12 ♀♀, 70 km S of Saynshand, 1100 m, 6.VIII.2007, leg. MH (PRC/MHC).

####### Distribution.

*Mongolia (Dornod, Dornogovi, Umnugovi); Kazakhstan (Rosa 2018).

####### Notes.

As supposed by Rosa (2018), living specimens are red and change to greenish after preparation.

###### Chrysis jaxartis

Taxon classificationAnimaliaHymenopteraChrysididae

Semenov, 1910

53D00E54-48D9-579F-9FED-E8D99843191A

Chrysis sybarita var. jaxartis Semenov-Tian-Shansky, 1910: 222. Lectotype ♂ (designated by Rosa et al. 2017a: 54). Kazakhstan: Djulek (Budapest) (examined) (*graelsii* group).

####### Material examined.

Mongolia: *Dornod*, 6 ♀♀, 100 km W of Choilbalsan, 820 m, 23.VII.2007, leg. MH (MHC); 9 ♀♀, 2 ♂♂, 20 km W of Choilbalsan, 800 m, 48°01'N, 114°14'E 24.VII.2007, leg. MH (MHC); *Khentii*, 11 ♀♀, 4 ♂♂, 100 km NE of Ondorkhaan, Kerulen River, 970 m, 22.VII.2007, leg. MH (MHC); *Selenge*, 3 ♀♀, 1 ♂, 90 km N of Ulaanbaatar, Segnez River, 1450 m, 6–8.VII.2003, leg. JH (MHC); *Tuv*, 5 ♀♀, 1 ♂, 50 km N of Ulaanbaatar, E of Mandal, 1180 m, 8–13.VIII.2007, leg. MH (MHC); 1 ♀, same date and locality, and collector (P. Tyrner priv. coll.); *Umnugovi*, 1 ♂, Gobi, Dalanzadgad, 25.VI.2003, leg. JH (MHC).

####### Distribution.

*Mongolia (Dornod, Khentii, Selenge, Tuv, Umnugovi); Asiatic-European, from Greece, Iran, and Turkey to Central Asia (Rosa et al. 2019).

###### Chrysis leptomandibularis

Taxon classificationAnimaliaHymenopteraChrysididae

Niehuis, 2000

3C8BE02B-C06D-575C-9780-9B1CF6CF652B

Chrysis leptomandibularis Niehuis, 2000: 192. Holotype ♀; Germany: Rheinland-Pfalz, Monsheim (Frankfurt) (ignita group).

####### Material examined.

Mongolia: *Tuv*, 3 ♀♀, 2 ♂♂, 50 km E of Ulaanbaatar, Tuul River, 22.VI.2003, leg. JH, det. J. van der Smissen and MH (MHC).

####### Distribution.

*Mongolia (Tuv); Asiatic-European from Europe to Russia (Rosa et al. 2019).

###### Chrysis mane

Taxon classificationAnimaliaHymenopteraChrysididae

Semenov, 1912

87D6DEE1-93D6-557C-80F8-762EB2D7D7E4

Chrysis mane Semenov-Tian-Shanskij, 1912: 192. Lectotype ♂ (designated by Bohart in Kimsey and Bohart 1991: 436); China: Alashan (192 (descr.), depository: ZIN).
Chrysis mane : Kimsey and Bohart 1991: 436 (China [not Mongolia]: Gansu, Quingai, cat., ignita group).

####### Material examined.

Mongolia: *Tuv*, 1 ♂, 50 km N of Ulaanbaatar, E of Mandal, 1180 m, 8–13.VIII.2007, leg. MK (PRC).

####### Distribution.

*Mongolia (Tuv); China (Gansu, Qinghai, Inner Mongolia) (Rosa et al. 2014).

###### Chrysis matutina

Taxon classificationAnimaliaHymenopteraChrysididae

Semenov, 1967

BE57E98A-32E1-5A22-8A53-6BCCE5D6A040

Chrysis (Tetrachrysis) matutina Semenov-Tian-Shanskij, 1967: 179. Holotype ♀; China: Gansu (ZIN) (ignita group).

####### Material examined.

Mongolia: *Arkhangai*, 1 ♂, 90 km NE of Tsetserleg, 48°03'N, 102°25'E, 27.VII.2005, leg. JH (MHC).

####### Distribution.

*Mongolia (Arkhangai); China (Gansu) (Rosa et al. 2014).

###### Chrysis mediata

Taxon classificationAnimaliaHymenopteraChrysididae

Linsenmaier, 1951

7E117F5F-8980-504D-89F1-8AC4547BE8EB

Chrysis ignita var. mediata Linsenmaier, 1951: 76. Lectotype ♀ (designated by Linsenmaier 1959: 154); Switzerland: Wallis (NMLS) (examined) (ignita group).

####### Material examined.

Mongolia: *Tuv*, 2 ♀♀, Ulaanbaatar Bog Duul, 11.VII.1983, leg. Karl Bleyl, det. Linsenmaier 1992 (NMLS).

####### Distribution.

*Mongolia (Tuv); Palaearctic region excluding Japan (Linsenmaier 1997b).

###### Chrysis mocsaryi

Taxon classificationAnimaliaHymenopteraChrysididae

Radoszkowski, 1889

6A0D935B-FC48-57B3-A16D-9E29DBD2CD34

Chrysis (Tetrachrysis) Mocsaryi Radoszkowski, 1889: 29. Holotype ♀; Mongolia: Kobden (Khovd) (ISEA-PAS) (examined) (*comparata* group). Mocsáry 1889: 426 (cat., descr., Mongolia).
Chrysis mocsaryi : Dalla Torre 1892: 78 (cat., Mongolia); Kimsey and Bohart 1991: 440 (cat., Mongolia: Kobden, *comparata*-scutellaris group). Rosa et al. 2015: 41 (cat., type series), 42 (Fig. 4).

####### Material examined.

Mongolia: Holotype ♀, golden rounded label, Kansu Kobden-Owatu 12/VIII [handwritten] *Mocsáry* [handwritten by Radoszkowski] // *Chrysis Mocsaryi* Rad. (tres interep.) [?] [handwritten by Mocsáry], label with right flagellum and metasoma, Mus. Pan Krakow [hadwritten by Dylewska].

####### Distribution.

Mongolia (Khovd) (Radoszkowski 1889).

###### Chrysis mysticalis

Taxon classificationAnimaliaHymenopteraChrysididae

Linsenmaier, 1959

076C949E-E9F3-5BFF-89EE-AC90C3C3EA13

Chrysis mysticalis Linsenmaier, 1959: 165. Holotype ♀; Spain: Zamora (Luzern) (examined) (*inaequalis* group).

####### Material examined.

Mongolia: *Arkhangai*, 1 ♀, 1 ♂, 90 km NE of Tsetserleg, 48°03'N, 102°25'E, 24.VII.2004, leg. MK (MHC); *Bulgan*, 2 ♂♂, Mongol Els Nat. Res., dunes, 47°24'N, 103°39'E, 31.VII.2005, leg. JH (MHC); *Dornogovi*, 1 ♀, 28 km of SE Chatan-Bulag, 3.VIII.2007, leg. MHMHC); *Tuv*, 1 ♂, 50 km E of Ulaanbaatar, Tuul River, 22.VI.2003, leg. JH (MHC); 2 ♀♀, 50 km N of Ulaanbaatar, E of Mandal, 1180 m, 8–13.VIII.2007, leg. MH (MHC); *Zavkhan*, 1 ♀, 1 ♂, 40 km SW of Uliastay, dunes, 18.VII.2005, leg. JH (MHC).

####### Distribution.

*Mongolia (Arkhangai, Bulgan, Dornogovi, Tuv, Zavkhan); from southern Europe to eastern Siberia (Rosa et al. 2017b).

###### Chrysis nox

Taxon classificationAnimaliaHymenopteraChrysididae

Semenov, 1954

949297CE-690B-5D5A-B643-5D23C246FD4D

Chrysis (Tetrachrysis) nox Semenov in Semenov-Tian-Shanskij and Nikol’skaya 1954: 128. Lectotype ♀ (designated by Bohart in Kimsey and Bohart 1991: 444); Tajikistan [not Mongolia]: Peter the Great Range, Yashil’-Kul’ Lake, 7.VIII.1911, leg. Golbek (ZIN) (examined) (*facialis* group). Rosa et al. 2017a: 42 (cat., type series), 158 (plate 97).
Chrysis nox : Kimsey and Bohart 1991: 444 (cat., Mongolia: Yihe Bogdo, Peter the Great Range, *facialis* group).

####### Material examined.

Mongolia: *Govi-Altai*, 4 ♀♀, 1 ♂, Ikhe-Bogdo, Gobi Altai, 30.VI–12.VII.1926, leg. P. Kozlov // Paratypes (ZIN); 4 ♀♀, idem, 15–17.VII.1926, Paratypes (ZIN); 1 ♀, North slope of Ikhe-Bogdo, 30.VI–12.VII.1926, leg. P. Kozlov, Paratypes (ZIN); 1 ♀, Ihe-Bogdo, Gob. Altai, 15–17.VII.1926, leg. P. Kozlov [in Cyrillic], det. M. Nikol’skaya (NMLS); *Tuv*, 1 ♀, Ulaanbaatar Bog Duul, 11.VII.1983, leg. Karl Bleyl, det. Linsenmaier 1990 (NMLS).

####### Distribution.

Mongolia (Govi-Altai, Tuv); Tajikistan (Rosa et al. 2017a).

###### Chrysis pavesii

Taxon classificationAnimaliaHymenopteraChrysididae

Rosa, 2017

61885804-0C34-59CA-9F6A-79EA9450938B

Chrysis pavesii Rosa in Rosa et al. 2017c: 27. Holotype ♀; Russia: Western Siberia, Altai Rep., 5 km SE of Chagan-Uzun, Tudtuyaryk River, 1780 m, 11.VII.2016, leg. M. Proshchalykin & V. Loktionov (ZIN) (examined) (*bihamata* group).

####### Material examined.

Mongolia: *Govi-Altai*, 1 ♂, 10 km SSE of Ich-Oba-Ula, 18.VII.1970, leg. E. Narchuk (ZIN).

####### Distribution.

*Mongolia (Govi-Altai, Tuv); Russia (western Siberia) (Rosa et al. 2017c).

###### Chrysis priapus

Taxon classificationAnimaliaHymenopteraChrysididae

Rosa, 2018

F6C71551-A6FE-58EA-9B54-9C5E032C3913

Chrysis priapus Rosa, 2018: 281. Holotype ♂; Mongolia: Govi-Altai Prov., 8 km SE of Argalant-Ula (ZIN) (examined) (slava group).

####### Material examined.

Mongolia: *Govi-Altai*, 1 ♂, 8 km SE of Argalant-Ula, 20.VI.1980, leg. G. Medvedev (ZIN).

####### Distribution.

Mongolia (Govi-Altai) (Rosa 2018).

###### Chrysis pseudobrevitarsis

Taxon classificationAnimaliaHymenopteraChrysididae

Linsenmaier, 1951

237AC968-D491-5D8E-BC76-281C06048A5D

Chrysis ignita var. pseudobrevitarsis Linsenmaier, 1951: 79. Lectotype ♀ (designated by Linsenmaier 1959: 158); Switzerland: Wallis (NMLS) (examined) (ignita group).
Chrysis (Chrysis) pseudobrevitarsis : Linsenmaier 1997b: 114 (descr., Mongolia, without locality, see in Material examined).
Chrysis pseudobrevitarsis : Rosa et al. 2019: 153 (cat., Mongolia).

####### Material examined.

Mongolia: *Arkhangai*, 1 ♂, 70 km NE of Tsetserleg, 25.VII.2005, leg. JH (MHC); *Tuv*, 1 ♀, Tereltz, 8.VII.1983, leg. Karl Bleyl, det. Linsenmaier 1992 (NMLS); 2 ♀♀, 50 km N of Ulaanbaatar, E of Mandal, 1180 m, 8–13.VIII.2007, leg. MH (MHC).

####### Distribution.

Mongolia (Arkhangai, Tuv); Asiatic-European, from western Europe to Mongolia (Linsenmaier 1997a).

###### Chrysis pupilla

Taxon classificationAnimaliaHymenopteraChrysididae

Semenov, 1967

7E381F1A-3960-520B-8FEE-EF644453A548

Chrysis (Tetrachrysis) pupilla Semenov-Tian-Shanskij, 1967: 174. Holotype ♀; Uzbekistan: Termez (ZIN) (examined) (*varidens* group).

####### Material examined.

Mongolia: *Umnugovi*, 1 ♀, “Yuzhno-Gobiyskiy Ajmag, sajr Undyn-Gol, 25 km S of Khan-Bogdo, 7.VII.1971”, leg. M. Kozlov (ZIN).

####### Distribution.

*Mongolia (Umnugovi); Uzbekistan (Semenov-Tian-Shanskij 1967).

###### Chrysis rutilans

Taxon classificationAnimaliaHymenopteraChrysididae

Olivier, 1791

E5B29A70-A3E3-5AFC-B1B3-123163EDDF94

Chrysis rutilans Olivier, 1791: 676. Type unknown; France: Angoumois (depository unknown) (splendidula group).

####### Material examined.

Mongolia: *Tuv*, 1 ♀, 50 km N of Ulaanbaatar, E of Mandal, 1180 m, 8–13.VIII.2007, leg. MH (MHC).

####### Distribution.

*Mongolia (Tuv); Palaearctic, from western Europe and North Africa to China and Japan (Linsenmaier 1997b).

###### Chrysis schencki

Taxon classificationAnimaliaHymenopteraChrysididae

Linsenmaier, 1968

7304B10D-C499-5D86-A27A-D9D906C9FCCC

Chrysis (Chrysis) ignita ssp. schenckiana Linsenmaier, 1959: 156, nom. praeocc., nec Mocsáry, 1912. Holotype ♀; Switzerland: Graubünden (Luzern) (examined) (ignita group).
Chrysis (Chrysis) ignita schencki Linsenmaier, 1968: 99. Replacement name for C. ignita schenckiana Linsenmaier, 1959.

####### Material examined.

Mongolia: *Arkhangai*, 2 ♀♀, Chuluut Gol River, 47°48'N, 100°19'E, 23.VII.2005, leg. JH, det. J. Van der Smissen (MHC); *Bulgan*, 2 ♀♀, Mongol Els Nat. Res., dunes, 47°24'N, 103°39'E, 31.VII.2005, leg. JH, det. J. Van der Smissen (MHC).

####### Distribution.

*Mongolia (Arkhangai, Bulgan); Asiatic-European, from western Europe to Central Asia, Siberia and Japan (Rosa et al. 2019).

###### Chrysis sibirica

Taxon classificationAnimaliaHymenopteraChrysididae

Rosa, 2017

C208046B-73F3-508C-AC60-9EE23C33F069

Chrysis sibirica Rosa in Rosa et al., 2017c: 24. Holotype ♀; Russia: Tuva Rep., 31 km NEE of Erzin, Erzin River, 18.vii.2014, leg. A. Lelej, M. Proshchalykin & V. Loktionov (St. Petersburg) (*bihamata* group).

####### Material examined.

Mongolia: *Arkhangai*, 1 ♀, Chuluut Gol River, 47°48'N, 100°19'E, 23.VII.2005, leg. JH (MHC).

####### Distribution.

*Mongolia (Arkhangai); Russia (Eastern Siberia) (Rosa et al. 2017c).

###### Chrysis solida

Taxon classificationAnimaliaHymenopteraChrysididae

Haupt, 1957

B106AAD1-4734-5029-99D9-B1B84C677B6E

Chrysis ignita solida Haupt, 1957: 115. Lectotype ♀ (designated by Niehuis 2000: 199); Poland: Bellinchen [= Bielinek] (MLU) (ignita group).
Chrysis mediata fenniensis Linsenmaier, 1959: Móczár 1967: 189 (cat., Mongolia: 1 ♀, Čojbalsan [= Dornod] aimag: Menengijn valley, 160 km W of Bujr nur Lake, 600 m, Exp. Dr. Z. Kaszab, 1965, nr. 416, 15.VIII.1965).

####### Material examined.

None examined.

####### Distribution.

Mongolia (Dornod); Asiatic-European, from western Europe to Japan (Linsenmaier 1997b).

###### Chrysis splendidula unica

Taxon classificationAnimaliaHymenopteraChrysididae

Radoszkowski, 1891

770ED8E5-784A-5F85-AE43-D999B483568D

Chrysis splendidula var. unica Radoszkowski, 1891: 189. Syntypes ♂, ♀; Turkmenistan: Ashgabad (ISEA-PAS) (examined) (splendidula group).

####### Material examined.

Mongolia: *Arkhangai*, 1 ♂, 90 km NE of Tsetserleg, 48°03'N, 102°25'E, 27.VII.2005, leg. JH (MHC).

####### Distribution.

*Mongolia (Arkhangai); Turkmenistan (Radoszkowski 1891).

###### Chrysis subcoriacea

Taxon classificationAnimaliaHymenopteraChrysididae

Linsenmaier, 1959

256A09E7-C5E4-5E29-A0E0-4878B2FCB50A

Chrysis (Chrysis) longula ssp. subcoriacea Linsenmaier, 1959: 160. Holotype ♀; Finland: Kyrkslätt [= Kirkkonummi] (Luzern) (examined) (ignita group).

####### Material examined.

Mongolia: *Arkhangai*, 1 ♂, 70 km NE of Tsetserleg, 25.VII.2005, leg. JH, det. J. Van der Smissen (MHC).

####### Distribution.

*Mongolia (Arkhangai); Asiatic-European, from western Europe to Central Asia, Russia and Japan (Rosa et al. 2019).

###### Chrysis viridula

Taxon classificationAnimaliaHymenopteraChrysididae

Linnaeus, 1761

261C71FD-11B5-51B4-96FA-284AFA77C85C

Chrysis viridula Linnaeus, 1761: 415. Type unknown; Sweden (unknown) (viridula group).

####### Material examined.

Mongolia: *Tuv*, 1 ♀, 100 km E of Ulaanbaatar, 20 km NE of Tereltz, Tuul River, 15–21.VII.2003, leg. JH (PRC).

####### Distribution.

*Mongolia (Tuv); Asiatic-European, from western Europe to Central Asia, Russia, and Japan (Rosa et al. 2019).

##### Genus *Chrysura* Dahlbom, 1845

*Chrysura* Dahlbom, 1845: 6. Type species: *Chrysis austriaca* Fabricius, 1804, by subsequent designation of Bodenstein 1939: 125.

###### Chrysura dichroa

Taxon classificationAnimaliaHymenopteraChrysididae

(Dahlbom, 1854)

299D26E3-082D-5C99-9399-2C4FF34C8CBC

Chrysis dichroa Dahlbom, 1854: 146. Lectotype ♀ (designated by Rosa and Xu 2015: 17); Hungary: Budapest (MSNT) (dichroa group).

####### Material examined.

Mongolia: *Zavkhan*, 1 ♀, 40 km SW of Uliastay, dunes, 18.VII.2005, leg. JH (MHC).

####### Distribution.

*Mongolia (Zavkhan); Asiatic-European, from western Europe to Central Asia and western Siberia (Rosa et al. 2019).

###### Chrysura ignifrons

Taxon classificationAnimaliaHymenopteraChrysididae

Brullé, 1833

F682CC3D-ADAB-51C8-A32D-DFD7C2531284

Chrysis ignifrons Brullé, 1833: 375. Holotype ♂ [not ♀]; Greece: Peloponnese (Paris) (examined) (austriaca group).

####### Material examined.

Mongolia: *Zavkhan*, 1 ♂, 40 km SW of Uliastay, dunes, 18.VII.2005, leg. JH (MHC).

####### Distribution.

*Mongolia (Zavkhan); Palaeartcic, from southern Europe and northern Africa to Middle East and Central Asia (Rosa et al. 2019).

##### Genus Euchroeus Latreille, 1809

*Euchroeus* Latreille, 1809: 49. Type species: *Chrysis purpurata* Fabricius, 1787 [= *Euchroeus purpuratus* (Fabricius, 1787)], by monotypy.

###### Euchroeus mongolicus

Taxon classificationAnimaliaHymenopteraChrysididae

Tsuneki, 1947

58F1AEE0-8E40-53F1-9E4E-4301DC131A36

Euchroeus purpuratus f. mongolicus Tsuneki, 1947: 54. Holotype ♀; China: Inner Mongolia: Apaka (NIAS).
Euchroeus (Euchroeus) mongolicus : Linsenmaier 1959: 73 (tax., descr., Mongolia [= Inner Mongolia]), 200 (fig. 213).
Spinolia (Euchroeus) par Semenov, 1967: 189 (cat., Mongolia: 1 ♀, Uburchangaj aimag: Changaj Mt., 8 km W of Somon Chajrchandulaan, 2000 m, Exp. Dr. Z. Kaszab, 1964, nr. 217, 28.VI.1964; 1 ♀, Southgobi aimag: 60 km E of Somon Bulgan, 1120 m, Exp. Dr. Z. Kaszab 1964, nr. 262, 4.VII.1964).
Brugmoia quadrata f. mongolica : Kimsey and Bohart 1991: 296 (cat., China [not Mongolia]: Apaka).
Euchroeus mongolicus : Rosa et al. 2014: 68 (cat.), 111 (Plate 59); Rosa et al. 2019: 326 (Mongolia, figs 83, 84).

####### Material examined.

Mongolia: *Govi-Altai*, 25 ♀♀, 1 ♂, 70 km E of Altay city, Guulin, 14.VII.2005, leg. JH (MHC, PRC).

####### Distribution.

Mongolia (*Govi-Altai, Umnugovi, Uvurkhangai); China (Inner Mongolia, Shanxi) (Rosa et al. 2014).

###### Euchroeus orientis

Taxon classificationAnimaliaHymenopteraChrysididae

Semenov, 1910

CBC93850-32D7-5A45-B075-7A5AC6976BD3

Pseudochrysis (Euchroeus) purpurata subsp. orientis Semenov-Tian-Shansky, 1910: 214. Lectotype ♂, designated by Kimsey in Kimsey & Bohart 1991: 296; China: Bugas near Khami, SE of Tian-Shan [China, Xinjiang], 3–5.IX.1895, leg. V. Roborovskij & P. Kozlov (ZIN) (examined).
Spinolia (Euchroeus) orientis : Móczár 1967: 189 (cat., Mongolia: 1 ♂, Suchebaator [= Sukhbaatar] aimag: Ongon elis, 10 km S of Somon Chongor, 900 m, Exp. Dr. Z. Kaszab, 1965, nr. 356, 3.–4.VIII.1965; 1 ♂, 44 km SSW of Baruun urt, 1050 m, Exp. Dr. Z. Kaszab, 1965, nr. 349, 2.–3.VIII.1965).
Euchroeus (Euchroeus) purpuratus orientis : Linsenmaier 1968: 46 (descr., Mongolia, observed in the collections of HNHM and MHNH, without precise localities).

####### Material examined.

None examined.

####### Distribution.

Mongolia (Sukhbaatar); China (Xinjiang) (Rosa et al. 2014).

##### Genus *Pentachrysis* Lichtenstein, 1876

*Pentachrysis* Lichtenstein, 1876: 227. Type species: *Chrysis amoena* Eversmann 1858 [= *Pentachrysis amoena* (Eversmann, 1858)], by subsequent designation of Ashmead 1902: 226

###### Pentachrysis amoena

Taxon classificationAnimaliaHymenopteraChrysididae

(Eversmann, 1858)

72011E11-19C8-5705-849B-1F2D3F1481EC

Chrysis amoena Eversmann, 1858: 562. Holotype ♀; Russian SFSR: ‘campis transuralensibus’ (ISEA-PAS) (examined).
Pentachrysis amoena : Kimsey and Bohart 1991: 521 (Mongolia, without specific locality); Rosa et al. 2019: 197 (cat., Mongolia).

####### Material examined.

None examined.

####### Distribution.

Mongolia (without locality); Asiatic-European, from eastern Europe to Mongolia (Kimsey and Bohart 1991).

##### Genus *Pseudochrysis* Semenov, 1891

*Pseudochrysis* Semenov, 1891: 444. Type species: *Chrysura humboldti* Dahlbom, 1845: 6 [= *Pseudochrysis humboldti* (Dahlbom, 1845)], by subsequent designation of Semenov 1892: 485.

*Pseudospinolia* Linsenmaier, 1951: 65 (as subgenus of *Euchroeus* Latreille, 1809). Type species: *Chrysis uniformis* Dahlbom, 1854: 149, by original designation. Synonymized by Rosa et al. 2017b

###### Pseudochrysis gengiskhan

Taxon classificationAnimaliaHymenopteraChrysididae

Rosa, 2017

73DDA6E3-505B-5632-89FE-F2339B3E73E8

Pseudochrysis gengiskhan Rosa in Rosa et al. 2017c: 9. Holotype ♀; Mongolia: Övörkhangay [Bulgan], 137 km NE of Aravaykheer, 47°20'N, 103°40.5'E, 1250 m, 26.vii.2004, leg. J. Halada (ZIN) (examined). Rosa et al. 2019: 198 (cat., Mongolia), 328 (fig. 91).

####### Material examined.

Mongolia: *Bayankhongor*, 1 ♀, 1 ♂, 129 km NW of Bayankhongor, 47°13'N, 99°55'E, 2590 m, 16.VII.2004, leg. JH (MHC); *Bulgan*, 4 ♀♀, Mongolia, 137 km NE of Aravaykheer, 47°20'N, 103°40.5'E, 1250 m, 26.VII.2004, leg. JH (PRC/ZIN); 1 ♂, Mongol Els Nat. Res., dunes, 47°24'N, 103°39'E, 31.VII.2005, leg. JH (MHC); *Dornod*, 2 ♂♂, 100 km W of Choibalsan, 820 m, 23.VII.2007, leg. MH (MHC); 3 ♂♂, 20 km W of Choibalsan, 48°01'N, 114°14'E, 800 m, 24.VII.2007, leg. MH (MHC); 2 ♂♂, 50 km SW of Choibalsan, 960 m, 25.VII.2007, leg. JH (MHC); *Selenge*, 2 ♂♂, 90 km N of Ulaanbaatar, Segnez River, 1450 m, 6–8.VII. 2003, leg. JH (MHC); *Sukhbaatar*, 1 ♂, 200 km SSE of Baruun-Urt, Moltsoy Els, 1250 m, 27.VII.2007, Allotype, leg. MK (ZIN); 3 ♂♂, ibid, 27.VII.2007, leg. MH (MHC); 2 ♀♀, 7 ♂♂, 100 km SSW of Baruun-Urt, 1100 m, 30.VII.2007, leg. MH (MHC); *Tuv*, 4 ♂♂, Khangayn Mts, 5 km N of Khunt, 20.VII.2005, leg. JH (MHC); 4 ♂♂, 75 km W of Ulaanbaatar, dunes, 2.VIII.2005, leg. JH (MHC); 13 ♀♀, 50 km N of Ulaanbaatar, E of Mandal, 1180 m, 8–13.VIII.2007, leg. JH (MHC); 8 ♀♀, ibid, leg. MH (MHC).

####### Distribution.

Mongolia (*Bayankhongor, *Dornod, *Selenge, Sukhbaatar, *Tuv, Bulgan); Russia (Siberia) (Rosa et al. 2017c).

###### Pseudochrysis neglecta

Taxon classificationAnimaliaHymenopteraChrysididae

(Shuckard, 1837)

62F88D4F-437D-55AB-B8EB-DA74F2DB614F

Chrysis neglecta Shuckard, 1837: 169. Lectotype ♀ (designated by Morgan 1984: 9); England (LSL).

####### Material examined.

Mongolia: *Tuv*, 1 ♂, 50 km E of Ulaanbaatar, Tuul River, 22.VI.2003, leg. JH (MHC).

####### Distribution.

*Mongolia (Tuv); Holarctic: from west Europe to Turkey, Siberia, Manchuria and Russian Far East (Rosa et al. 2019); North America (Bohart and Kimsey 1982).

##### Genus *Spinolia* Dahlbom, 1854

*Spinolia* Dahlbom, 1854: 363. Type species: *Spinolia magnifica* Dahlbom, 1854 [= *Spinolia lamprosoma* (Förster, 1853)], by monotypy.

###### Spinolia spinosa

Taxon classificationAnimaliaHymenopteraChrysididae

Rosa & Halada sp. nov.

688E8405-0A11-500A-ABD2-9A33A2377945

http://zoobank.org/A105F4B1-87F4-4005-B1A0-09844A7247B0

**Figure 5.**
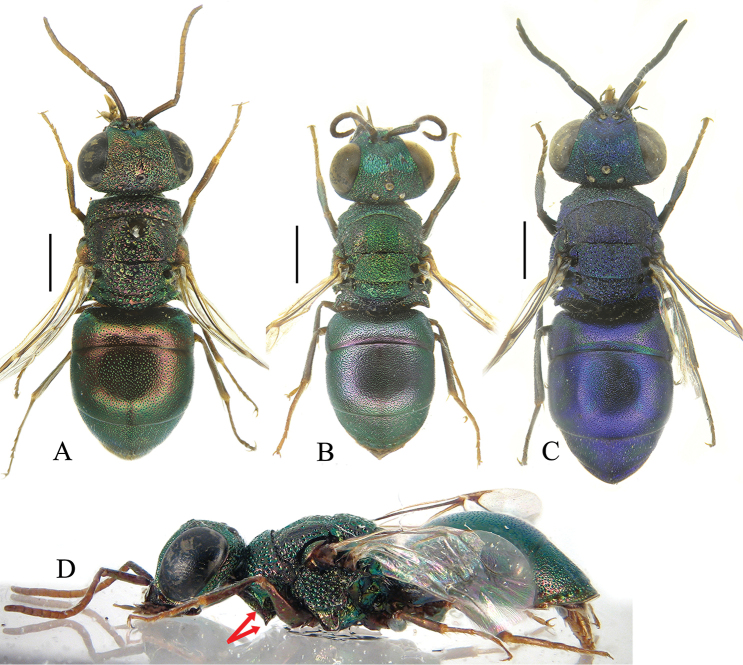
*Spinolia* species, females **A***S. spinosa* sp. nov., dorsal view **B***S. chalcites*, dorsal view **C***S. unicolor*, dorsal view **D***Spinolia spinosa* sp. nov., lateral view: arrows pointing at pronotal and propleural spines. Scale bars: 1.0 mm.

**Figure 6.**
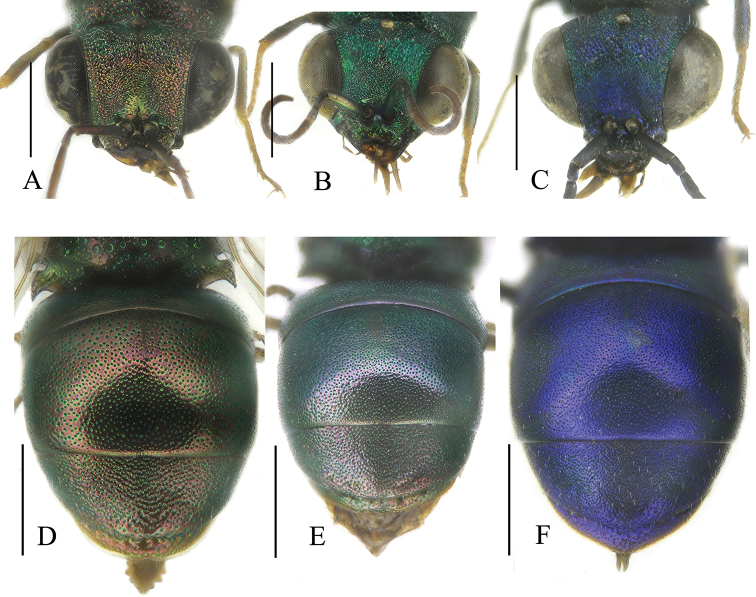
*Spinolia* species, females, head in frontal view (**A–C**), and metasoma in dorsal view (**D–F**). **A***S. spinosa* sp. nov. **B***S. chalcites***C***S. unicolor*. **D***S. spinosa* sp. nov. **E***S. chalcites***F***S. unicolor*. Scale bars: 1.0 mm.

Figures 5A, D , 6A, D

####### Type material.

***Holotype***: ♀, Mongolia: *Bayankhongor*, Edringiyn-Nuru Ridge, 100 km SSW of Bayan-Under, 5.IX.1970, leg. V. Zaitzev (ZIN).

####### Diagnosis.

*Spinolia spinosa* sp. nov. is closely related to Central Asian species of the *unicolor* group, which includes *S. chalcites* (Mocsáry, 1890), *S. rusalka* (Semenov, 1901), *S. hedychroides* (Bingham, 1903) and other small species so far considered synonyms of *S. chalcites* (Kimsey and Bohart 1991). *S. spinosa* sp. nov. female can be easily separated from all these species by: lateral pronotal area and propleuron ventrally V-shaped carinate, displaying two teeth in lateral view (Fig. 5D) (vs. unmodified in other species); mesopleuron with large and deep scrobal sulcus subtended by large projecting subrectangular carina (Fig. 5D) (vs. U-shaped carina); sparse, deep and large punctures on mesosoma (Fig. 6D), and sparse and deep punctures on metasoma (vs. punctation with dense, shallow and tiny punctures on mesosoma, denser and shallower on metasoma); antennae yellowish, distinctly elongate (Fig. 5A) (vs. black to dark brown, with short to very short flagellomeres); head, in frontal view, transversely subrectangular (Fig. 6A) and not triangular (Fig. 6B); with bulging eyes, similarly to *S. unicolor*. It is additionally separated from *S. unicolor* by punctation, elongate and yellowish antennae and bronze body colour (entirely blue body in *S. unicolor*, with shortened, blackish flagellomeres).

####### Description.

**Female.** Body length 6.0 mm. Fore wing length 3.8 mm. OOL = 2.3 MOD; POL = 1.9 MOD; MS = 0.7 MOD; relative length of P:F1:F2:F3 = 1.0:1.4:1.0:0.8; subantennal space: 1.4 MOD. ***Head*.** Vertex with deep and contiguous punctures, as large as 0.25 MOD; vertex moderately depressed and impunctate in front of anterior ocellus and impunctate laterad of posterior ocelli; median anterior depression developed to upper scapal basin; TFC faint; frons continuous, without two flattened or concave, striate areas; scapal basin almost flat, laterally densely micro-punctate, medially with contiguous punctures forming transverse winkles (Fig. 6A); lower part of scapal basin medially impunctate and sulcate; apex of clypeus discoloured, W-shaped and bent under, medially the folded part measures 0.6 MOD. Malar space very short, distinctly less than 1 MOD. Antennae elongate, with flagellomeres as long as 1.5 × their width. Mouth parts elongate (as long as 0.8 × head length) and evidently protruding from oral fossa. ***Mesosoma*.** Pronotal groove barely visible; anterolateral corner of the pronotum projected to form an acute humeral angle (Fig. 5A); lateral pronotal area ventrally V-shaped carinate forming an acute tooth (Fig. 5D); propleuron ventrally carinate in a large V-shaped tooth (Fig. 5D). Mesosoma punctation dorsally with large, spaced punctures; interspaces medially polished, laterally micro-punctate; notauli incomplete, visible and deep only basally towards the transscutal fissure; parapsidal furrows fully visible; mesopleuron with a large subrectangular area subtended the mesepimeron + mesepisternum; posterior propodeal projections narrow, acute and downward directed. Wing venation unmodified, with long Rs bending slightly away from costal margin, leaving marginal cell broadly open. ***Metasoma*.** Punctation on T1 with tiny, sparse punctures (separated by 1–4 PD) (Fig. 6D), laterally micro-punctate on interspaces; T2 with larger and deeper punctures, anterodorsally denser (0.1–2 PD), laterally micro-punctate on interspaces; T3 with coarse to contiguous small punctures; T3 pit row barely sunken, with small, round pits, equally spaced; posterior pit row area almost polished, with a few, sparse, tiny punctures; T3 with two lateral angles and fully bordered by hyaline margin. Metasomal invaginated T5, T6, and S5 with several dorsal and lateral lobes. S2 black spots oval, transversally placed and separated 0.5 MOD each other. ***Colouration*.** Body coppery-bronze, darker to black on median area of mesoscutum; ventrally golden to copper; tegulae golden to non-metallic yellowish on outer margin; tarsi dark brown. Mandible brown, lighter medially. Scape and pedicel coppery, antennomeres yellowish-orange, darker on distal segments. Legs pale coloured, with slight metallic reflections, with non-metallic proximal and distal joints; tarsi yellowish. Forewings hyaline, slightly amber, with light brown veins. ***Vestiture***. Whitish, short and sparse setae on head and mesosoma (up to 1.5 MOD long); face with short whitish setae (less than 1.0 MOD); metasoma with short (less than 1. MOD) whitish, sparse setae on T3 and ventrally on S2 and S3 and femora.

**Male.** Unknown.

####### Etymology.

The specific epithet *spinosa* (feminine) is derived from the Latin adjective *spinosus* (thorny) for the long and acute teeth ventrally displayed on pronotum and propleuron and clearly visible in lateral view (Fig. 5D).

####### Distribution.

Mongolia (Bayankhongor).

###### Spinolia unicolor

Taxon classificationAnimaliaHymenopteraChrysididae

(Dahlbom, 1831)

EEFB9EE8-9503-51AA-B34A-B12B7D9AA051

Chrysis unicolor Dahlbom, 1831: 32. Syntypes ♂♂; Sweden: Scania: Lomma and Käflinge [= Kävlinge] (ZMUL) (examined).
Spinolia unicolor : Kimsey and Bohart 1991: 552 (cat., Mongolia, without locality).

####### Material examined.

Mongolia: *Govi-Altai*, 1 ♂, 70 km E of Altay city, Guulin, 14.VII.2005, leg. JH (MHC).

####### Distribution.

Mongolia (*Govi-Altai); Asiatic-European: from eastern Europe to Mongolia (Linsenmaier 1959, Kimsey and Bohart 1991).

##### Genus *Stilbum* Spinola, 1806

*Stilbum* Spinola, 1806: 9. Type species: *Chrysis calens* Fabricius, 1781, by subsequent desigation of Latreille 1810: 437.

###### Stilbum calens

Taxon classificationAnimaliaHymenopteraChrysididae

(Fabricius, 1781)

D018A140-B734-5F89-8B9A-0990943D0C66

Chrysis calens Fabricius, 1781: 455. Holotype ♀; Russia: Siberia (NHMUK).
Stilbum calens zimmermanni Linsenmaier, 1959: Linsenmaier 1997b: 132 (China, Inner Mongolia [not Mongolia]).

####### Material examined.

Mongolia: *Arkhangai*, 9 ♀♀, 25 km NE of Tsetserleg, 47°38'N, 101°45'E, 23.VII.2004, leg. JH (MHC); *Bulgan*, 1 ♀, Mongol Els Nat. Res., dunes, 47°24'N, 103°39'E, 31.VII.2005, leg. JH (MHC); *Dornogovi*, 1 ♂, 28 km SE of Chatan-Bulag, steppe, 3.VIII.2007, leg. MK (PRC); *Tuv*, 2 ♀♀, 1 ♂, Khangayn Mts, 5 km N of Khunt, 20.VII.2005, leg. JH (MHC); 1 ♂, 75 km W of Ulaanbaatar, dunes, 2.VIII.2005, leg. JH (MHC); *Uvurkhangai*, 3 ♀♀, 12 km E of Aravaykheer, 46°22'N, 102°49'E, 1800 m, 3.VII.2004, leg. JH (MHC).

####### Distribution.

*Mongolia (Arkhangai, Bulgan, Dornogovi, Tuv, Uvurkhangai); widely distributed in the Palaearctic Region (Tsuneki 1948, Linsenmaier 1959), Russia (Siberia), China (Liaoning, Beijing, Inner Mongolia, Shanxi) (Rosa et al. 2019). Linsenmaier (1997b) mentioned Mongolia in the distribution range of *Stilbum calens*, yet the specimen examined by the Swiss author was collected in China, Inner Mongolia: 1 ♂, Hutjertu Gol [currently Khujirt Gol River, near Bailingmiaozhen monastery, N of Baotou, Inner Mongolia, China] 1927, Sven Hedins Exp. Ctr. Asien Dr. Hummel, det. Linsenmaier 1963 (NMLS).

##### Genus *Trichrysis* Lichtenstein, 1876

*Trichrysis* Lichtenstein, 1876: 27. Type species: *Sphex cyanea* Linnaeus, 1758 [= *Trichrysis cyanea* (Linnaeus, 1758)], by monotypy.

###### Trichrysis cyanea

Taxon classificationAnimaliaHymenopteraChrysididae

(Linnaeus, 1758)

3664516F-0F76-52A0-B871-62051D404D5F

Sphex cyanea Linnaeus, 1758: 572. Lectotype ♂ (designated by Morgan 1984: 10); Europe (LSL).

####### Material examined.

Mongolia: *Bayankhongor*, 1 ♀, 16 km SW of Bayankhongor, 46°13'N, 100°30'E, 2165 m, 10.VII.2004, leg. JH (MHC); *Selenge*, 1 ♀, 90 km N of Ulaanbaatar, Segnez River, 1450 m, 6–8.VII.2003, leg. JH (MHC); *Tuv*, 1 ♀, 100 km E of Ulaanbaatar, 20 km NE Tereltz, Tuul River, 15–21.VII.2003, leg. JH (MHC); 2 ♀♀, 50 km N of Ulaanbaatar, E of Mandal, 1180 m, 8–13.VIII.2007, leg. MH (MHC).

####### Distribution.

*Mongolia (Bayankhongor, Selenge, Tuv); Palaearctic, from western Europe and northern Africa to Central Asia, China and Japan (Rosa et al. 2019).

###### Trichrysis pellucida

Taxon classificationAnimaliaHymenopteraChrysididae

(du Buysson, 1887)

1C358ADE-4B45-5CE2-B464-CA9C792790D5

Chrysis pellucida du Buysson, 1887: 183. Lectotype ♀ (designated by Rosa et al. 2016: 123); China (MNHN) (examined).
Chrysis (Trichrysis) buyssoni Mocsáry, 1889: 323. Unnecessary replacement name for Chrysis pellucida du Buysson, 1887.
Chrysis (Trichrysis) mongolica Mocsáry, 1914: 24. Lectotype ♀ (designated by Bohart in Kimsey and Bohart 1991: 571); Mongolia (HMNH).

####### Material examined.

Mongolia: 1 ♀, *mongolica* Mocs. typ. det. Mocsáry, red label, *Chrysis* L. *pellucida* Buyss. Linsenmaier det. 59, Lectotype *Chrysis mongolica* Mocs. ♀ RM Bohart, id nr. 135554 HNHM Hym. coll. Paralectotypes: 4 ♀♀, Mongolia, *mongolica* Mocs. typ. det. Mocsáry, red label, *Chrysis* L. *pellucida* Buyss. Linsenmaier det. 59, Paralectotype *Chrysis mongolica* Mocs. ♀ RM Bohart, id nr. 135555–135558 HNHM Hym. coll.

####### Distribution.

Mongolia (without locality); East-Palaearctic: Russia (Far East), China (Liaoning, Inner Mongolia, Hebei, Beijing, Hunan) (Rosa et al. 2016, 2019).

###### Trichrysis secernenda

Taxon classificationAnimaliaHymenopteraChrysididae

(Mocsáry, 1912)

846820A8-4737-54AA-A4D8-2A90DB6C28E1

Chrysis (Trichrysis) secernenda Mocsáry, 1912: 376. Lectotype ♂ (designated by Bohart in Bohart and French 1986: 342); Uzbekistan: Gouldsha (HNHM) (examined).

####### Material examined.

Mongolia: *Selenge*, 1 ♂, 90 km N of Ulaanbaatar, Segnez River, 1450 m, 6–8.VII.2003, leg. JH (MHC).

####### Distribution.

*Mongolia (Selenge); Afghanistan, Uzbekistan, China (Xinjiang, Ningxia) (Rosa et al. 2016).

#### Tribe Elampini

##### Genus *Colpopyga* Semenov, 1954

*Colpopyga* Semenov, 1954: 137. Type species: *Hedychrum flavipes* Eversmann, 1858 [= *Colpopyga flavipes* (Eversmann, 1858)], by original designation.

###### Colpopyga nesterovi

Taxon classificationAnimaliaHymenopteraChrysididae

Rosa, 2017

2C119BA7-224A-5337-90D9-A3A7D6D7DF01

Colpopyga nesterovi Rosa, 2017b: 301. Holotype ♀; Kazakhstan: Aktobe Prov., Mugodzhary Mt., Emba River valley, 17.vi.1985, leg. M. Nesterov (ZIN) (examined).

####### Material examined.

Mongolia: *Dornod*, 2 ♀♀, 1 ♂, 20 km W of Choilbalsan, 800 m, 48°01'N, 114°14'E, 24.VII.2007, leg. MH (MHC).

####### Distribution.

*Mongolia (Dornod); Kazakhstan (Rosa 2017b).

##### Genus *Elampus* Spinola, 1806

*Elampus* Spinola, 1806: 10. Type species: *Chrysis panzeri* Fabricius, 1804 [= *Elampus panzeri* (Fabricius, 1804)], by subsequent designation of Latreille 1810: 437.

*Ellampus* Agassiz, 1846: 136. Unjustified emendation of *Elampus* Spinola, 1806 (part.).

*Notozus* Förster, 1853: 351. Type species: *Notozus frivaldszkii* Förster, 1853 [= *Elampus spina* (Lepeletier, 1806)], by subsequent designation of Ashmead 1902: 228.

###### Elampus albipennis

Taxon classificationAnimaliaHymenopteraChrysididae

(Mocsáry, 1889)

049AE56C-772D-5084-9B17-B3684BFCC398

Ellampus (Notozus) albipennis Mocsáry, 1889: 80. Lectotype ♂ (designated by Móczár 1964: 447); Russia: Astrakhan (HMNH) (examined).

####### Material examined.

Mongolia: *Arkhangai*, 93 ♀♀, 30 ♂♂, Chuluut Gol River, 47°48'N, 100°19'E, 23.VII.2005, leg. JH (MHC); *Dornogovi*, 1 ♂, Orgon, 11.VII.2005, leg. JH (MHC).

####### Distribution.

*Mongolia (Arkhangai, Dornogovi); Asiatic-European, from eastern Europe, Saudi Arabia, UAE to Central Asia and eastern Siberia (Rosa et al. 2019).

###### Elampus coloratus

Taxon classificationAnimaliaHymenopteraChrysididae

Rosa, 2017

15084A1B-CE65-5126-9378-90CD870E62C2

Elampus coloratus Rosa in Rosa et al. 2017d: 2. Holotype ♂; Russia: Tyva Rep., 20 km SSW of Erzin, Tore-Khol’ Lake (ZIN) (examined).

####### Material examined.

Mongolia: *Sukhbaatar*, 1 ♂, Lun-Ula, 30 km WSW of Dariganga, 1.VII.1971, leg. I. Kerzhner (ZIN).

####### Distribution.

Mongolia (Sukhbaatar); Russia (Tyva Rep.) (Rosa et al. 2017d).

###### Elampus montanus

Taxon classificationAnimaliaHymenopteraChrysididae

(Mocsáry, 1890)

6486FD88-10D0-55F2-8AB1-693AB14437AC

Ellampus (Notozus) montanus Mocsáry, 1890: 49. Holotype ♂; Turkey: Buyuk Agri Dagi (Mount Ararat) (ISEA-PAS) (examined).

####### Material examined.

Mongolia: *Dornogovi*, 6 ♂♂, Orgon, 11.VII.2005, leg. JH (MHC).

####### Distribution.

*Mongolia (Dornogovi); Turkey (Mocsáry 1890) and Central Asia (unpubl. data).

###### Elampus panzeri

Taxon classificationAnimaliaHymenopteraChrysididae

(Fabricius, 1804)

502428A1-21F3-5429-85F3-203C18346F6D

Chrysis scutellaris Panzer, 1798: fig. 51, pl. 11. Type unknown; Germany: Nürnberg (depository unknown), nom. praeocc., nec Fabricius, 1794.
Chrysis panzeri Fabricius, 1804: 172. Replacement name for Chrysis scutellaris Panzer, 1798.

####### Material examined.

Mongolia: *Arkhangai*, 1 ♂, 25 km NE of Tsetserleg, 47°38'N, 101°45'E, 23.VII.2004, leg MK (MHC); *Zavkhan*, 1 ♀, 2 ♂♂, 40 km SW of Uliastay, dunes, 18.VII.2005, leg. JH (MHC).

####### Distribution.

*Mongolia (Arkhangai, Zavkhan); Asiatic-European, from western Europe to eastern Siberia, Russian Far East, and China (Heilongjiang) (Rosa et al. 2019).

###### Elampus sanzii

Taxon classificationAnimaliaHymenopteraChrysididae

Gogorza, 1887

7CD44450-8295-5AB2-8411-2D5AD5BAD52C

Elampus sanzii Gogorza, 1887: 33. Holotype ♂; Spain: Madrid (MCNM).
Notozus sanzii : Móczár 1967: 183 (cat., Mongolia: Central aimag, Zuun-Chara, 850 m, Exp. Dr. Z. Kaszab, 1964, Nr. 281; 8.VII.1964).

####### Material examined.

None examined.

####### Distribution.

Mongolia (Tuv); Asiatic-European, from Iberian Peninsula to Mongolia and eastern Siberia (Rosa et al. 2019).

###### Elampus spinifemoris

Taxon classificationAnimaliaHymenopteraChrysididae

(Móczár, 1967)

CB1F0770-9B73-5930-8278-23A5197DFD1F

Notozus spinifemoris Móczár, 1967: 185. Holotype ♀; Mongolia: Uvurkhangai aimag: Arc Bogd ul, ca. 20 km S of von Somon Chovd, 1760 m, Exp. Dr. Z. Kaszab, 1964, 22.VI.1964 (HNHM) (examined). Rosa et al. 2017e: 113 (cat., typ., Mongolia: Arc Bogd ul, fig. 87).

####### Material examined.

Mongolia: *Uvurkhangai*, 1 ♀, Uburchangaj aimag, Arc Bogd ul, cca 20 km S of von somon Chovd, 1760 m Exp. Dr. Z. Kaszab, 1964, Nr. 170, 22.VI.1964, *Notozus* sp. nov. ♀ det. Móczár 965, ♀ *Omalus* Pz. *Notozus panzeri* F. Linsenmaier det. 1964, Holotype ♀ *Notozus spinifemoris* L. Móczár 1966, Hym. Typ. No. 87 Mus. Budapest, id nr. 134892 HNHM Hym. coll. (HNHM).

####### Distribution.

Mongolia (Uvurkhangai) (Móczár 1967).

##### Genus *Hedychridium* Latreille, 1802

*Hedychridium* Abeille de Perrin, 1878: 3. Type species: *Hedychrum minutum* Lepeletier, 1806 [= *Hedychridium ardens* (Coquebert, 1801)], by subsequent designation of Ashmead 1902: 227.

###### Hedychridium ardens

Taxon classificationAnimaliaHymenopteraChrysididae

(Coquebert, 1801)

B1965D70-88B2-5844-A04D-9F09C697D48D

Chrysis ardens Coquebert, 1801: 59. Holotype ♀; France: Bordeaux (MNHN?).
Hedychridium ardens : Móczár 1967: 189 (cat., Mongolia: Uvurkhangai aimag: Baga Bogd ul, between Somon Bogd and Somon Baruun Hajan-ulaan, 1900 m, Exp. Dr. Z. Kaszab, 1964, nr. 176, 23.VI.1964).

####### Material examined.

Mongolia: *Arkhangai*, 1 ♀, 90 km NE of Tsetserleg, 48°03'N, 102°25'E, 24.VII.2004, leg. JH (MHC); *Bayankhongor*, 9 ♀♀, 8 ♂♂, 16 km SW of Bayankhongor, 46°13'N, 100°30'E, 2165 m, 10.VII.2004, leg. JH (MHC); *Dornod*, 2 ♀♀, 3 ♂♂, 100 km W of Choilbalsan, 820 m, 23.VII.2007, leg. MH (MHC); 5 ♀♀, 2 ♂♂, 20 km W of Choilbalsan, 800 m, 48°01'N, 114°14'E, 24.VII.2007, leg. MH (MHC); *Khentii*, 1 ♀, 100 km NE of Ondorkhaan, Kerulen River, 970 m, 22.VII.2007, leg. MH (MHC); *Selenge*, 2 ♀♀, 90 km N of Ulaanbaatar, Segnez River, 1450 m, 6–8.VII.2003, leg. JH (MHC); *Sukhbaatar*, 1 ♀, 1 ♂, 100 km SSW of Baruun-Urt, 1100 m, 30.VII.2007, leg. MH (MHC); *Ulaanbaatar*, 7 ♀♀, Ulaanbaatar, Tuul River valley, 12.VII.2003, leg. JH (MHC); *Zavkhan*, 2 ♂♂, 40 km SW of Uliastay, dunes, 18.VII.2005, leg. JH (MHC).

####### Distribution.

Mongolia (*Arkhangai, *Bayankhongor, *Dornod, *Khentii, *Selenge, *Sukhbaatar, *Ulaanbaatar, Uvurkhangai, *Zavkhan); Asiatic-European, from Europe and Middle East to Russia (Far East) (Kimsey and Bohart 1991; Kurzenko and Lelej 2007).

###### Hedychridium asianum

Taxon classificationAnimaliaHymenopteraChrysididae

Linsenmaier, 1997

D2CECE62-6B04-5D59-A6ED-53CE40D63EDD

Hedychridium integrum ssp. asianum Linsenmaier, 1997a: 254. Holotype ♂, Mongolia: Ulan Bator, 1900 m (UKC).

####### Material examined.

Mongolia: *Arkhangai*, 1 ♂, 90 km NE of Tsetserleg, 48°03'N, 102°25'E, 24.VII.2004, leg. JH (MHC); *Bayankhongor*, 1 ♂, 16 km SW of Bayankhongor, 46°13'N, 100°30'E, 2165 m, 10.VII.2004, leg. JH (MHC); *Bulgan*, 3 ♂♂, 137 km NE of Aravaykheer, 47°20'N, 103°45.5'E, 1250 m, 2.VII.2004, leg. JS (MHC); 2 ♀♀, 2 ♂♂, 143 km NE of Aravaykheer, 47°24'N, 103°39'E, 1300 m, 26.VII.2004, leg. MH (MHC); *Ulaanbaatar*, 1 ♂, Ulaanbaatar, 16.VII.1989, 1900 m, leg. Peter Salk, det. Linsenmaier, 1997 (NMLS).

####### Distribution.

Mongolia (*Arkhangai, *Bayankhongor, *Bulgan, Ulaanbaatar); China (Gansu) (Rosa et al. 2014).

####### Remarks.

*Hedychridium asianum* was described as a subspecies of *H. integrum*. As recently pointed out by Paukkunen et al. (2014), *H. integrum* is a synonym of *H. ardens* and *H. integrum* sensu Linsenmaier (1959) is *H. cupreum*.

###### Hedychridium belokobylskiji

Taxon classificationAnimaliaHymenopteraChrysididae

Rosa, 2017

74DD55EE-4F99-5D9C-A1CC-71EACCDE496D

Hedychridium belokobylskiji Rosa in Rosa et al. 2017d: 11. Holotype ♀; Russia: Eastern Siberia, Tuva Rep., 12 km SW of Samagaltai, Dyttyg-Khem River, 19.VII.2014, leg. A. Lelej, M. Proshchalykin, V. Loktionov (ZIN) (examined).

####### Material examined.

Mongolia: *Tuv*, 1 ♀, 50 km N of Ulaanbaatar, E of Mandal, 1180 m, 8–13.VIII.2007, leg. MK (PRC).

####### Distribution.

*Mongolia (Tuv); Russia (Eastern Siberia) (Rosa et al. 2019).

###### Hedychridium cupreum

Taxon classificationAnimaliaHymenopteraChrysididae

(Dahlbom, 1845)

5CBA7E09-D20F-5506-8563-6C0F4E0E3283

Hedychrum cupreum Dahlbom, 1845: 3. Lectotype ♀ (designated by Paukkunen et al. 2014: 23); Sweden: Lund (NHMW) (examined).

####### Material examined.

Mongolia: *Bayankhongor*, 2 ♀♀, 16 km SW of Bayankhongor, 46°13'N, 100°30'E, 2165 m, 10.VII.2004, leg. JH (MHC); *Dornogovi*, 1 ♂, 65 km SE of Chatan-Bulag, 1020 m, 2.VIII.2007, leg. MH (MHC); *Govi-Altai*, 4 ♀♀, 70 km E of Altay City, Guulin, 14.VII.2005, leg. JH (MHC); *Tuv*, 2 ♀♀, 1 ♂, 50 km E of Ulaanbaatar, Tuul River, 22.VI.2003, leg. JH (MHC); 1 ♀, Khangaun Mts, 5 km N of Khunt, 20.VII.2005, leg. JH (MHC); *Umnugovi*, 3 ♀♀, Gobi, 100 km SW of Dalanzadgad, Bayanzag, 1–2.VII.2003, leg. JH (MHC); *Uvurkhangai*, 1 ♂, 12 km E of Aravaykheer, 46°22'N, 102°49'E, 1800 m, 3.VII.2004, leg. JH (MHC); *Zavkhan*, 2 ♀♀, 1 ♂, 40 km SW of Uliastay, dunes, 18.VII.2005, leg. JH (MHC).

####### Distribution.

*Mongolia (Bayankhongor, Dornogovi, Govi-Altai, Tuv, Umnugovi, Uvurkhangai, Zavkhan); Asiatic-European, from north-western Europe to Mongolia and China (Rosa et al. 2014).

####### Remarks.

Specimens from Mongolia display an unusual red colouration.

###### Hedychridium gabriellae

Taxon classificationAnimaliaHymenopteraChrysididae

Rosa, 2017

339A9801-E63D-58CF-A7C4-C614B90B745D

Hedychridium gabriellae Rosa in Rosa et al. 2017d: 19. Holotype ♀; Russia: Eastern Siberia, Tuva Rep., 20 km of SSW Erzin, Tore-Khol’ Lake, 30.VI–3.VII.2013, leg. V. Loktionov & M. Proshchalykin (ZIN) (examined).

####### Material examined.

Mongolia: *Bayankhongor*, 18 ♂♂, 22 ♀♀, 75 km S of Bayankhongor, 45°20'N, 100°48.5'E, 1330 m, 8.VII.2004, leg. JH, JS (MHC); *Dornogovi*, 5 ♀♀, 5 ♂♂, 65 km SE of Chatan-Bulag, 1020 m, 2.VIII.2007, leg. MH (MHC); *Tuv*, 1 ♂, 70 km W of Ulaanbaatar, 1070 m, dunes, 16.VIII.2007, leg. MH (MHC).

####### Distribution.

*Mongolia (Bayankhongor, Dornogovi, Tuv); Russia (Eastern Siberia) (Rosa et al. 2019).

###### Hedychridium longigena

Taxon classificationAnimaliaHymenopteraChrysididae

Rosa, 2017

C4414CEE-E8DE-5093-9652-CBFAABC69B44

Hedychridium longigena Rosa in Rosa et al. 2017d: 21. Holotype ♀; Russia: Irkutsk Prov., 8 km N of Irkutsk, Angara River, sandy slopes, 10.VII.2001, collector unknown (ZIN) (examined).

####### Material examined.

Mongolia: *Bayankhongor*, 1 ♀, 1 ♂, 56 km NW of Bayankhongor, 46°33'N, 100°12'E, 2200 m, 12.VII.2004, leg. JS (MHC); *Bulgan*, 2 ♀♀, 137 km NE of Aravaykheer, 47°20'N, 103°45.5'E, 1250 m, 2.VII.2004, leg. MK (MHC); 3 ♀♀, ibid, 26.VII.2004, JH (MHC); *Dornod*, 1 ♂, 100 km W of Choilbalsan, 820 m, 23.VII.2007, leg. MH (MHC); *Dornogovi*, 1 ♂, 65 km SE of Chatan-Bulag, 1020 m, 2.VIII.2007, leg. MH (MHC); *Khentii*, 2 ♀♀, 2 ♂♂, 100 km NE of Ondorkhaan, Kerulen River, 970 m, 22.VII.2007, leg. MH (MHC); *Selenge*, 1 ♀, 90 km N of Ulaanbaatar, Segnez River, 1450 m, 6–8.VII.2003, leg. JH (MHC); *Tuv*, 10 ♀♀, 9 ♂♂, 50 km N of Ulaanbaatar, E of Mandal, 1180 m, 8–13.VIII.2007, leg. MK (PRC); 4 ♂♂, 70 km W of Ulaanbaatar, 1070 m, dunes, 16.VIII.2007, leg. MH (MHC).

####### Distribution.

*Mongolia (Bayankhongor, Bulgan, Dornod, Dornogovi, Khentii, Selenge, Tuv); Russia (Eastern Siberia) (Rosa et al. 2019).

###### Hedychridium propodeale

Taxon classificationAnimaliaHymenopteraChrysididae

Rosa, 2017

18B1546C-4CE5-5BCB-A5B3-1885747B0B11

Hedychridium propodeale Rosa in Rosa et al. 2017d: 16. Holotype ♀; Russia: Eastern Siberia, Tuva Rep., 20 km SSW of Erzin, Tore-Khol’ Lake, 3.VII.2013, leg. V. Loktionov & M. Proshchalykin (ZIN) (examined).

####### Material examined.

Mongolia: *Govi-Altai*, 1 ♀, Mongolia W, 70 km E of Altay city, Guulin, 14.VII.2005, leg. JH (MHC).

####### Distribution.

*Mongolia (Govi-Altai); Russia (Eastern Siberia) (Rosa et al. 2019).

###### Hedychridium roseum

Taxon classificationAnimaliaHymenopteraChrysididae

(Rossi, 1790)

83478E37-22EE-54CF-86C3-395A84137EBA

Chrysis carnea var. rosea Rossi, 1790: 75. Syntypes; Italy (Berlin?).

####### Material examined.

Mongolia: *Arkhangai*, 1 ♂, 90 km NE of Tsetserleg, 48°03'N, 102°25'E, 24.7.2004, leg. JH (MHC); *Dornod*, 21 ♀♀, 4 ♂♂, 100 km W of Choilbalsan, 820 m, 23.VII.2007, leg. MH (MHC); 19 ♀♀, 1 ♂, 20 km W of Choilbalsan, 800 m, 48°01'N, 114°14'E, 24.7.2007, leg. MH (MHC); *Dornogovi*, 4 ♂♂, 2 km SE of Khuvsgol, 5.VIII.2007, leg. MH (MHC); *Sukhbaatar*, 1 ♀, 1 ♂, 100 km SSW of Baruun-Urt, 1100 m, 30.VII.2007, leg. MH (MHC); 4 ♂♂.

####### Distribution.

*Mongolia (Arkhangai, Dornod, Dornogovi, Sukhbaatar); Asiatic-European, from western Europe to Russia (Far East) (Rosa et al. 2019).

####### Remarks.

The record from Korea (Tsuneki 1953b and Korean checklists) must be referred to *Hedychridium tsunekii* Linsenmaier, 1959. In fact, Linsenmaier (1959) described as *Hedychridium tsunekii* the Korean specimens collected and identified by Tsuneki (1953) as *H. roseum*.

##### Genus *Hedychrum* Latreille, 1802

*Hedychrum* Latreille, 1802: 317. Type species: *Chrysis lucidula* Fabricius, 1775 [= *Hedychrum nobile* (Scopoli, 1763)], by monotypy.

###### Hedychrum chalybaeum

Taxon classificationAnimaliaHymenopteraChrysididae

Dahlbom, 1854

E2991175-823B-5B88-B08F-BFEC4A9FBC84

Hedychrum chalybaeum Dahlbom, 1854: 64. Syntypes ♂♂; Europe: ‘Europa media et meridionali’, Russia, Prussia, Silesia (MfN, ZMUL) (examined). Móczár 1967: 188 (cat., Mongolia: 1 ♀, Sukhbaatar aimag: 44 km SSW of Baruun urt, 1050 m, Exp. Dr. Z. Kaszab, 1965, nr. 349, 2.–3.VIII.1965; 1 ♀, Chadatin-bulan, 60 km N of Somon Bajanterem, 950 m, Exp. Dr. Z. Kaszab, 1965, nr. 340, 31.VII.1965).

####### Material examined.

Mongolia: *Bayankhongor*, 1 ♂, 2 km S of Bayankhongor, 46°12'N, 100°43'E, 1800 m, 10.VII.2004, leg. JH (PRC); 20 ♀♀, 2 ♂♂, 56 km NW of Bayankhongor, 46°33'N, 100°12'E, 2200 m, 12.VII.2004, leg. JH (PRC); *Dornod*, 16 ♀♀, 9 ♂♂, 100 km W of Choilbalsan, 820 m, 23.VII.2007, leg. MH (MHC); 16 ♀♀, 6 ♂♂, 20 km W of Choilbalsan, 800 m, 48°01'N, 114°14'E 24.VII.2007, leg. MH (MHC); 1 ♂, 15 km W of Choibalsan, Kerulen River, 770 m, 24.VII.2007, leg. MK (PRC); 1 ♂, 50 km SW of Choibalsan, 960 m, 25.VII.2007, leg. JH (MHC); 1 ♀, 50 km SW of Choibalsan, 960 m, 25.VII.2007, leg. MH (MHC); *Govi-Altai*, 2 ♂♂, 70 km E of Altay city, Guulin, 14.VII.2005, leg. JH (MHC); *Selenge*, 1 ♂, 90 km N of Ulaanbaatar, Segnez River, 1450 m, 6–8.VII.2003, leg. JH (PRC); *Sukhbaatar*, 6 ♀♀, 2 ♂♂, 100 km SSW of Baruun-Urt, 1100 m, 30.VII.2007, leg. MH (MHC); *Tuv*, 1 ♂, 50 km E of Ulaanbaatar, Tuul River, 22.VI.2003, leg. JH (MHC); *Ulaanbaatar*, 1 ♂, Ulaanbaatar, Tuul River valley, 12.VII.2003, leg. JH (PRC); 1 ♀, 7 km E of Ulaanbaatar, Gachuurt, 47°55'N, 107°06'E, 31.VII.2002, 1310 m, leg. JS (PRC).

####### Distribution.

Mongolia (Bayankhongor, *Dornod, *Govi-Altai, *Selenge, Sukhbaatar, Tuv, *Ulaanbaatar); widely distributed in the Palaearctic Region (Linsenmaier 1959; Kurzenko and Lelej 2007), China (Heilongjiang, Inner Mongolia, Gansu) (Rosa et al. 2014).

###### Hedychrum gerstaeckeri

Taxon classificationAnimaliaHymenopteraChrysididae

Chevrier, 1869

4FA957BD-3FFE-5603-8C54-EAD079F88868

Hedychrum gerstaeckeri Chevrier, 1869: 47. Syntypes ♀♀, ♂♂, [not holotype]; Switzerland (Geneva) (examined).

####### Material examined.

Mongolia: *Khentii*, 3 ♀♀, 100 km NE of Ondorkhaan, Kerulen River, 970 m, 22.VII.2007, leg. MH (MHC); *Selenge*, 1 ♀, 5 ♂♂, 90 km N of Ulaanbaatar, Segnez River, 1450 m, 6–8.VII.2003, leg. JH (MHC); *Tuv*, 4 ♀♀, 50 km N of Ulaanbaatar, E of Mandal, 1180 m, 8–13.VIII.2007, leg. MH (MHC).

####### Distribution.

*Mongolia (Khentii, Selenge, Tuv); Palaearctic and Oriental region, from western Europe to Russian Far East, Japan, China and Taiwan (Rosa et al. 2014, 2019).

###### Hedychrum lama

Taxon classificationAnimaliaHymenopteraChrysididae

du Buysson, 1891

98F28AEC-29A3-5A91-9FF7-2EAC92978EB9

Hedychrum lama du Buysson, 1891: 31. Lectotype ♂ (designated by Kimsey in Kimsey and Bohart 1991: 215); Mongolia: Kansu-Kobden Owatu (MNHN).

####### Material examined.

Mongolia: *Khovd*, 1 ♂, Mongolie O. Radoszkowsky, Kansu-Kobden Owatu, 12.8, Mongolie Coll. R. du Buysson 1900, Type, Museum Paris.

####### Distribution.

Mongolia (Khovd) (du Buysson 1891).

###### Hedychrum longicolle

Taxon classificationAnimaliaHymenopteraChrysididae

Abeille de Perrin, 1877

B975A0F8-264A-5644-A238-43EF3DD6BBC8

Hedychrum longicolle Abeille de Perrin, 1877: 65. Lectotype ♀ (designated by Kimsey 1986: 108); France: Marseille, Toulon (Geneva, Paris) (examined).

####### Material examined.

Mongolia: *Bulgan*, 1 ♂, 170 km W of Ulaanbaatar, dunes, 1070 m, 16.VIII.2007, leg. MH (MHC); *Dornod*, 1 ♂, 50 km SW of Choibalsan, 960 m, 25.VII.2007, leg. JH (MHC); *Sukhbaatar*, 1 ♂, 200 km SSE of Baruun-Urt, Moltsoy Els, 1250 m, 27.VII.2007, leg. MH (MHC); 2 ♂♂, 6 ♀♀, 100 km SSW of Baruun-Urt, 1100 m, 30.VII.2007, leg. MH (MHC); *Tuv*, 1 ♀, 75 km W Ulaanbaatar, dunes, 2.VIII.2005, leg. JH (MHC); *Umnugovi*, 1 ♀, Gobi Gurvansaikhan National Park, 44°00'N, 101°50'E, 10.VII.2005, leg. JH (MHC).

####### Distribution.

*Mongolia (Bulgan, Dornod, Sukhbaatar, Tuv, Umnugovi); Palaearctic, from southern Europe and northern Africa, to western Asia, Siberia, and China (Rosa et al. 2019).

###### Hedychrum nobile

Taxon classificationAnimaliaHymenopteraChrysididae

(Scopoli, 1763)

EA24A491-3936-5BB7-8D89-3F4109B386E0

Sphex nobile Scopoli, 1763: 297. Holotype ♀; Italy [not Austria] (lost).

####### Material examined.

Mongolia: *Arkhangai*, 1 ♀, 25 km NE of Tsetserleg, 47°38'N, 101°45'E, 23.VII.2004, leg. JH (MHC); 4 ♂♂, 90 km NE of Tsetserleg, 48°03'N, 102°25'E, 24.VII.2004, leg. JH (MHC); 4 ♀♀, 9 ♂♂, ibid, 27.VII.2005, leg. JH (MHC); *Selenge*, 2 ♀♀, 12 ♂♂, 90 km N of Ulaanbaatar, Segnez River, 1450 m, 6–8.VII.2003, leg. JH (MHC); *Tuv*, 2 ♀♀, 50 km N of Ulaanbaatar, E of Mandal, 1180 m, 8–13.VIII.2007, leg. MH (MHC); *Zavkhan*, 3 ♂♂, 40 km SW of Uliastay, dunes, 18.VII.2005, leg. JH (MHC).

####### Distribution.

*Mongolia (Arkhangai, Selenge, Tuv, Zavkhan); Asiatic-European, from western Europe to Siberia (Rosa et al. 2019).

###### Hedychrum rutilans ermak

Taxon classificationAnimaliaHymenopteraChrysididae

Semenov, 1967

82CFF819-5B80-53B1-BFA2-87CAF48DD9E1

Hedychrum intermedium ermak Semenov-Tian-Shanskij, 1967: 142. Holotype ♂; Russia: Siberia, Shira Lake [Khakass Rep.], 24.VII.1897, Yu. Wagner (ZIN) (examined). Móczár 1967: 188 (cat., Mongolia: Sukhbaatar aimag: Ongon elis, 10 km S of Somon Chongor, 900 m, Exp. Dr. Z. Kaszab, 1965, nr. 357, 3.–4.VIII.1965).

####### Material examined.

Mongolia: *Arkhangai*, 1 ♀, 90 km NE of Tsetserleg, 48°03'N, 102°25'E, 24.VII.2004, leg. JH (MHC); 1 ♀, 2 ♂♂, 90 km NE of Tsetserleg, 48°03'N, 102°25'E, 27.VII.2005, leg. JH (MHC); *Dornod*, 2 ♂♂, 20 km W of Choibalsan, 48°01'N, 114°14'E, 800 m, 24.VII.2007, leg. MH (MHC); *Selenge*, 1 ♂, 90 km N of Ulaanbaatar, Segnez River, 1450 m, 6–8.VII.2003, leg. JH (MHC); *Tuv*, 1 ♀, 50 km N of Ulaanbaatar, E of Mandal, 1180 m, 8–13.VIII.2007, leg. MK (PRC); 7 ♀♀, 3 ♂♂, ibid, 8–13.VIII.2007, leg. MH (MHC).

####### Distribution.

Mongolia (*Arkhangai, *Dornod, *Selenge, Sukhbaatar, *Tuv); Russia (Siberia, Far East) (Rosa et al. 2019).

##### Genus *Holopyga* Dahlbom, 1845

*Holopyga* Dahlbom, 1845: 4. Type species: *Holopyga amoenula* Dahlbom, 1845, by subsequent designation of Ashmead 1902: 227.

###### Holopyga generosa asiatica

Taxon classificationAnimaliaHymenopteraChrysididae

Trautmann, 1926

7A268874-78C1-5ED1-8276-6C68833AB6C7

Holopyga gloriosa var. asiatica Trautmann, 1926: 5. Holotype ♀; Turkey: İzmir prov.: Smyrna (MFN) (examined).

####### Material examined.

Mongolia: *Selenge*, 3 ♀♀, 5 ♂♂, 90 km N of Ulaanbaatar, Segnez River, 1450 m, 6–8.VII.2003, leg. JH (MHC).

####### Distribution.

*Mongolia (Selenge); Asiatic-European, from southern Europe to China (Rosa et al. 2019).

###### Holopyga kaszabi

Taxon classificationAnimaliaHymenopteraChrysididae

Móczár, 1967

ED915242-2305-599A-92B6-74F7EDDFDADD

Holopyga kaszabi Móczár, 1967: 187. Holotype ♂; Mongolia: Ostgobi aimag 40 km NW of Chara-Eireg 1150 m Exp. Dr. Z. Kaszab, 1963 (HNHM) (examined). Rosa et al. 2017e: 108 (cat., type series, Mongolia).

####### Material examined.

Mongolia: *Dornogovi*, 1 ♂, Ostgobi aimag 40 km NW of Chara-Eireg, 1150 m Exp. Dr. Z. Kaszab, 1963, Nr. 62, 30.VI.1963, Holotype ♂ *Holopyga kaszabi* n. sp. det. Móczár 1966, Hym. Typ. No. 89, id nr. 134927 HNHM Hym. coll. (HNHM); 1 ♀, Ostgobi aimag 40 km NW of Chara-Eireg 1150 m Exp. Dr. Z. Kaszab, 1963, Nr. 62, 30.VI.1963, Allotype ♀ *Holopyga kaszabi* n. sp. det. Móczár 1966, Hym. Typ. No. 96, id nr. 134933 HNHM Hym. Coll. (HNHM). 1 ♂: Ostgobi aimag 40 km NW of Chara-Eireg 1150 m Exp. Dr. Z. Kaszab, 1963, Nr. 62, 30.VI.1963, *Holopyga* sp. n. ? <handwritten by Móczár>, ♂ Allotype *Holopyga* Dhlb. *diversicolor* Lins. Linsenmaier 1966, Paratype ♂ *Holopyga kaszabi* n. sp. det. Móczár 1966, Hym. Typ. No. 90 (HNHM); 1 ♂, Mongolia, Ostgobi aimag 40 km NW of Chara-Eireg 1150 m Exp. Dr. Z. Kaszab, 1963, Nr. 62, 30.VI.1963, Paratype ♂ *Holopyga kaszabi* n. sp. det. Móczár 1966, Hym. Typ. No. 91, id nr. 134929 HNHM Hym. coll. (HNHM); 1 ♂: Mongolia, Ostgobi aimag 40 km NW of Chara-Eireg 1150 m Exp. Dr. Z. Kaszab, 1963, Nr. 62, 30.VI.1963, Paratype ♂ *Holopyga kaszabi* n. sp. det. Móczár 1966, Hym. Typ. No. 93, id nr. 134930 HNHM Hym. coll. (HNHM); 1 ♂, Mongolia, Ostgobi aimag 40 km NW of Chara-Eireg 1150 m Exp. Dr. Z. Kaszab, 1963, Nr. 62, 30.VI.1963, Paratype ♂ *Holopyga kaszabi* n. sp. det. Móczár 1966, Hym. Typ. No. 94, id nr. 134931 HNHM Hym. coll. (HNHM); 1 ♀: Mongolia, Ostgobi aimag 40 km NW of Chara-Eireg 1150 m Exp. Dr. Z. Kaszab, 1963, Nr. 62, 30.VI.63, Holopyga gloriosa ? intermedia ? det. L. Móczár, Type *Holopyga* Dhlb. *diversicolor* Lins. Linsenmaier 1966, Paratype ♀ *Holopyga kaszabi* n. sp. det. Móczár 1966, Hym. Typ. No. 95 / id nr. 134932 HNHM Hym. coll. (HNHM); 1 ♂, Mongolia, Catgobi aimag 40 Km NW of Chara-Eireg 1150 m, Exp.Dr. Z. Kaszab, 1963, Nr.62, 30.VI.1963, Paratype (NMLS); 1 ♀, Mongolia, Catgobi aimag 40 Km NW of Chara-Eireg, 1150 m, Exp. Dr. Z. Kaszab, 1963 / Nr.62, 30.VI.1963, Paratype (NMLS); *Umnugovi*, 1 ♂, Gobi, Dalanzadgad, 25.6.2003, leg. JH (MHC); *Uvurkhangai*, 1 ♂, 139 km SW of Aravaykheer, 45°17'N, 101°41'E, 1430 m, 4.VII.2004, leg. JS (MHC).

####### Distribution.

Mongolia (Dornogovi, *Umnugovi, *Uvurkhangai) (Móczár 1967).

###### Holopyga minuma

Taxon classificationAnimaliaHymenopteraChrysididae

Linsenmaier, 1959

B223D605-87A7-598A-B882-8FD677537D97

Holopyga minuma Linsenmaier, 1959a: 31. Holotype ♀; Turkey: Niğde prov.: Niğde (NMLS) (examined).

####### Material examined.

Mongolia: *Dornod*, 22 ♀♀, 14 ♂♂, 100 km W of Choilbalsan, 820 m, 23.VII.2007, leg. MH (MHC); 25 ♀♀, 6 ♂♂, 20 km W of Choilbalsan, 800 m, 48°01'N, 114°14'E 24.VII.2007, leg. MH (MHC); *Sukhbaatar*, 1 ♂, 200 km SSE of Baruun-Urt, Moltsoy Els, 1250 m, 27.VII.2007, leg. MH (MHC).

####### Distribution.

*Mongolia (Dornod, Sukhbaatar); Asiatic-European, from central Europe to eastern Siberia (Rosa et al. 2019).

##### Genus *Omalus* Panzer, 1801

*Omalus* Panzer, 1801: 13. Type species: *Chrysis aenea* Fabricius, 1787, by monotypy.

###### Omalus aeneus

Taxon classificationAnimaliaHymenopteraChrysididae

(Fabricius, 1787)

F4EAF101-00E8-5CC4-B51B-64BD794EECD0

Chrysis aenea Fabricius, 1787: 284. Holotype ♀; Germany: Hala Saxonum [= Halle] (Copenhagen) (examined).

####### Material examined.

Mongolia: *Tuv*, 1 ♀, 50 km E of Ulaanbaatar, Tuul River, 22.VI.2003, leg. JH (MHC); *Ulaanbaatar*, 1 ♀, Ulaanbaatar, Tuul River valley, 12.VII.2003, leg. JH (MHC).

####### Distribution.

*Mongolia (Tuv, Ulaanbaatar); Holarctic and Oriental: from Europe and North Africa to Japan and Taiwan (Wei et al. 2014). Probably accidentally introduced to North America (Kimsey and Bohart 1991).

###### Omalus berezovskii

Taxon classificationAnimaliaHymenopteraChrysididae

(Semenov, 1932)

47569CF4-012C-5877-831B-8F8249F440C4

Ellampus (Dictenulus) berezovskii Semenov-Tian-Shanskij, 1932: 12. Holotype ♀; China: Sichuan (ZIN) (examined).

####### Material examined.

Mongolia: *Ulaanbaatar*, 1 ♂, Ulaanbaatar, Tuul River valley, 12.VII.2003, leg. JH (MHC).

####### Distribution.

*Mongolia (Ulaanbaatar); East-Palaearctic: Russia (Eastern Siberia, Far East), China (Ningxia, Sichuan) (Rosa et al. 2019).

###### Omalus margianus

Taxon classificationAnimaliaHymenopteraChrysididae

(Semenov, 1932)

E31AB170-F5C6-5FBD-BDAF-87400BD505B7

Ellampus (Dictenulus) margianus Semenov-Tian-Shanskij, 1932: 15. Lectotype ♀ (designated by Kimsey 1986: 107); Turkmenistan: Imam-baba (ZIN) (examined).

####### Material examined.

Mongolia: *Arkhangai*, 1 ♀, 90 km NE of Tsetserleg, 48°03'N, 102°25'E, 27.VII.2005, leg. JH (MHC); *Bayankhongor*, 1 ♀, 75 km S of Bayankhongor, 45°20'N, 100°48.5'E, 1330 m, 8.VII.2004, leg. JS (MHC); *Bulgan*, 2 ♂♂, 137 km NE of Aravaykheer, 47°20'N, 103°45.5'E, 1250 m, 2.VII.2004, leg. JH (MHC); 1 ♀, 1 ♂, 143 km NE of Aravaykheer, 47°24'N, 103°39'E, 1300 m, 26.VII.2004, leg. MH (MHC); 2 ♂♂, Mongol Els Nat. Res., dunes, 47°24'N, 103°39'E, 31.VII.2005, leg. JH (MHC); *Sukhbaatar*, 2 ♀♀, 100 km SSW of Baruun-Urt, 1100 m, 30.VII.2007, leg. MH (MHC); *Tuv*, 1 ♂, 80 km W of Ulaanbaatar, 1230 m, dunes, 17.VIII.2007, leg. MH (MHC).

####### Distribution.

*Mongolia (Arkhangai, Bayankhongor, Bulgan, Sukhbaatar, Tuv); Central Asia (Kimsey and Bohart 1991).

###### Omalus miramae

Taxon classificationAnimaliaHymenopteraChrysididae

(Semenov, 1932)

F908B839-05A2-5BC7-B4CD-7AFEDCB1C90D

Ellampus (Dictenulus) miramae Semenov-Tian-Shanskij, 1932: 13. Lectotype ♀ (designated by Rosa et al. 2017a: 76); Turkmenistan: Pereval (ZIN) (examined).

####### Material examined.

Mongolia: *Bayankhongor*, 1 ♂, 75 km S of Bayankhongor, 45°20'N, 100°48.5'E, 1330 m, 8.VII.2004, leg. JH (MHC); *Dornogovi*, 2 ♀♀, Orgon, 11.VII.2005, leg. JH (MHC); *Sukhbaatar*, 2 ♀♀, 200 km SSE of Baruun-Urt, Moltsoy Els, 1250 m, 27.VII.2007, leg. MH (MHC).

####### Distribution.

*Mongolia (Bayankhongor, Dornogovi, Sukhbaatar); Central Asia (Kimsey and Bohart 1991).

###### Omalus stella

Taxon classificationAnimaliaHymenopteraChrysididae

(Semenov, 1932)

4305C1CA-26A7-5417-B7CB-603E941ABDC8

Ellampus (Ellampus) stella Semenov-Tian-Shanskij and Nikol’skaya, 1954: 93. Lectotype ♀ (designated by Kimsey 1986: 107); Tajikistan: Stalinabad (currently Dushambe) (ZIN) (examined).

####### Material examined.

Mongolia: *Arkhangai*, 1 ♀, 90 km NE of Tsetserleg, 48°03'N, 102°25'E, 24.VII.2004, leg. JH (MHC); *Tuv*, 3 ♂♂, 75 km W of Ulaanbaatar, dunes, 2.VIII.2005, leg. JH (MHC); *Uvurkhangai*, 1 ♀, 159 km of SW Aravaykheer, 45°11'N, 101°26'E, 1250 m, 5.VII.2004, leg. JH (MHC).

####### Distribution.

*Mongolia (Arkhangai, Tuv, Uvurkhangai); Central Asia (Kimsey and Bohart 1991).

##### Genus *Philoctetes* Abeille de Perrin, 1879

*Philoctetes* Abeille de Perrin, 1879: 27. Type species: *Holopyga cicatrix* Abeille de Perrin, 1879 [= *Philoctetes micans* (Klug, 1835)], by subsequent designation of Ashmead 1902: 228.

*Ellampus* Agassiz, 1846: 136. Unjustified emendation of *Elampus* Spinola, 1806 (part.).

###### Philoctetes bogdanovii

Taxon classificationAnimaliaHymenopteraChrysididae

(Radoszkowski, 1877)

BDDA00C1-24C2-52AF-A99B-A0246391A210

Holopyga bogdanovii Radoszkowski, 1877: 5. Holotype ♂; Uzbekistan: Zarafshan (ZMMU) (examined).

####### Material examined.

Mongolia: *Arkhangai*, 1 ♂, 90 km NE of Tsetserleg, 48°03'N, 102°25'E, 27.7.2005, leg. JH (MHC).

####### Distribution.

*Mongolia (Arkhangai); Asiatic-European, from southern Europe, western Asia, Iran, and Turkey to Mongolia (Rosa et al. 2013; present record).

###### Philoctetes cynthiae

Taxon classificationAnimaliaHymenopteraChrysididae

Rosa, 2017

FECC811F-AE39-54B2-A96D-6DEC9D18E368

Philoctetes cynthiae Rosa in Rosa et al. 2017c: 35. Holotype ♀; Russia: Tyva Rep., 13 km SW of Samagaltai, Dyttyg-Khem River, 9.VII.2013, leg. V. Loktionov & M. Proshchalykin (ZIN) (examined).

####### Material examined.

Mongolia: *Bayankhongor*, 1 ♀, 1 ♂, 75 km S of Bayankhongor, 45°20'N, 100°48.5'E, 1330 m, 8.VII.2004, leg. JH (MHC); 1 ♀, 56 km NW of Bayankhongor, 46°33'N, 100°12'E, 2200 m, 12.VII.2004, leg. JS (MHC); 3 ♀♀, 7 ♂♂, 86 km NW of Bayankhongor, 46°50'N, 100°04'E, 2070 m, 14.VII.2004, leg. JH, MK (MHC); *Sukhbaatar*, 1 ♀, SE Mongolia, 200 km SSE of Baruun-Urt, Moltsoy Els, 1250 m, 27.VII.2007, leg. MK, paratype (PRC); 1 ♂, ibid, leg. MH (MHC); *Ulaanbaatar*, 2 ♂♂, Ulaanbaatar, Tuul River valley, 12.VII.2003, leg. JH (MHC); *Uvurkhangai*, 4 ♂♂, 12 km E of Aravaykheer, 46°22'N, 102°49'E, 1800 m, 3.VII.2004, leg. JH (MHC);

####### Distribution.

Mongolia (*Bayankhongor, Sukhbaatar, *Ulaanbaatar, *Uvurkhangai); Russia (Tyva Rep.) (Rosa et al. 2017c).

###### Philoctetes diakonovi

Taxon classificationAnimaliaHymenopteraChrysididae

(Semenov, 1932)

9A776651-90CF-5AA0-B0D9-9A731FE3E0AE

Ellampus (Ellampus) diakonovi Semenov-Tian-Shanskij, 1932: 34. Holotype ♀; Kazakhstan: “Turkestan septentr.: Bajgakum (ZIN) (examined).

####### Material examined.

Mongolia: *Dornogovi*, 1 ♀, 1 ♂, Orgon, 11.VII.2005, leg. JH (MHC).

####### Distribution.

*Mongolia (Dornogovi); Central Asia (Kimsey and Bohart 1991).

###### Philoctetes lyubae

Taxon classificationAnimaliaHymenopteraChrysididae

Rosa, 2017

94CBFEEC-2798-533A-A6C0-8004628A77F3

Philoctetes lyubae Rosa in Rosa et al. 2017c: 39. Holotype ♀; Russia: Tuva Rep., 20 km SSW of Erzin, Tore-Khol’ Lake, 3.VII.2013, leg. V. Loktionov & M. Proshchalykin (ZIN) (examined).

####### Material examined.

Mongolia: *Dornogovi*, 1 ♂, Atayn Mts, Gichigniv Nuruu, 10 km SW of Sain-Shand, 12.VII.2005, leg. JH (MHC).

####### Distribution.

*Mongolia (Dornogovi); Russia (East Siberia) (Rosa et al. 2017c).

###### Philoctetes mongolicus

Taxon classificationAnimaliaHymenopteraChrysididae

(du Buysson, 1901)

2E72708C-2D43-546D-B26E-0E43FB88D320

Ellampus horvathi var. mongolicus du Buysson, 1901: 98. Lectotype ♂ (designated by Móczár 1967: 186); N Mongolia (NHMW) (examined). Rosa et al. 2020: 87 (cat., type series), 88 (fig. 55).
Ellampus horwathi (!) var. mongolicus: Bischoff 1913: 8 (cat., North Mongolia).
Omalus (Notozus) mongolicus : Linsenmaier 1959: 23 (descr. Mongolia).
Omalus mongolicus : Móczár 1967: 186 (cat., Mongolia: 1 ♂, Uvurkhangai aimag: Arc Bogd ul, ca. 20 km S of Somon Chovd, 1760 m, Exp. Dr. Z. Kaszab, 1964, nr. 170, 22.VI.1964; 1 ♂, Ulan-Baator, Bogdo ul, 1500 m, Exp. Dr. Z. Kaszab, 1963, nr. 4, 16.VI.1963).
Philoctetes horvathi var. mongolicus : Kimsey and Bohart 1991: 256 (cat., North Mongolia).
Philoctetes mongolicus : Rosa et al. 2014: 33 (cat., distr.); 2015: 436 (cat., descr., tax., Mongolia: Ulaanbataar, Baruun-Urt Moltsoy Els).

####### Material examined.

Mongolia: *Arkhangai*, 1 ♂, 25 km NE of Tsetserleg, 47°38'N, 101°45'E, 23.VII.2004, leg. JH (MHC); *Bayankhongor*, 2 ♂♂, 16 km SW of Bayankhongor, 46°13'N, 100°30'E, 2165 m, 10.VII.2004, leg. JH (MHC); 1 ♂, 56 km NW of Bayankhongor, 46°33'N, 100°12'E, 2200 m, 12.VII.2004, leg. JH (MHC); 1 ♂, 86 km NW of Bayankhongor, 46°50'N, 100°04'E, 2070 m, 14.VII.2004, leg. JH (MHC); *Khentii*, 2 ♀♀, 100 km NE of Ondorkhaan, Kerulen River, 970 m, 22.VII.2007, leg. MH (MHC); *Selenge*, 1 ♀, 90 km N of Ulaanbaatar, Segnez River, 1450 m, 6–8.VII.2003, leg. JH (MHC); *Sukhbaatar*, 1 ♀, SE Mongolia, 200 km SSE of Baruun-Urt, Moltsoy Els, 1250 m, 27.VII.2007, MK (PRC); *Tuv*, 1 ♀, 50 km N of Ulaanbataar, E of Mandal, 1180 m, 8–13.VII.2007, leg. MK (PRC); 1 ♀, 1 ♂, ibid, leg. MH (MHC); *Uvurkhangai*, 1 ♂, N. Mongolei Leder 92, Ellampus Horvathi Mocs. var. mongolicus Buyss. var. nov. R. du Buysson det. 1901 ♂, Lectotype v. *mongolicus* Buysson det. L. Móczár <red label> (NHMW). Paralectotypes: 1 ♂ N. Mongolei Leder 92, Ellampus Horvathi Mocs. var. mongolicus Buyss. var. nov. R. du Buysson det. 1901 ♂, *Paralectotype* v. *mongolicus* Buysson det. L. Móczár <red label> (NHMW); 1 ♀, N. Mongolei Leder 92, Ellampus Horwathi Mocs. var. mongolicus Buyss. var. nov. R. du Buysson det. 1901 ♀, *Omalus horvathi* Mocs. det. L. Móczár (NHMW).

####### Distribution.

Mongolia (*Arkhangai, *Bayankhongor, *Khentii, *Sukhbaatar, *Tuv, Ulaanbaatar, Uvurkhangai); widely distributed from Mongolia to Central Asia and southern Russia to Volga (Trautmann 1927), China (Shanxi) (Rosa et al. 2014).

###### Philoctetes shokalskii

Taxon classificationAnimaliaHymenopteraChrysididae

(Semenov, 1932)

122EF46C-0557-5897-B18D-6EBF6EB68780

Ellampus (Dictenulus) shokalskii Semenov-Tian-Shanskij, 1932: 24. Lectotype ♀ (designated by Kimsey 1986: 107); Mongolia: “Mongolia borealis: prope oppid. [um] Urga [Ulaanbaatar], 1–4.VI.1909, leg. P. Kozlov (ZIN) (examined). Rosa et al. 2017a: 78 (cat., type series), 218 (Plate 218). Rosa et al. 2017e: 119 (Mongolia: Chentej aimak 10 km W von Somon Delgerchaan, 1250 m Exp. Dr. Z. Kaszab, 1965 // Nr. 476. 24.VIII.1965).
Omalus shokalskii : Móczár, 1967: 186 (cat., Mongolia: 1 ♂, Ostgobi aimag; 40 km NW of Chara-Eireg, 1150 m, Exp. Dr. Z. Kaszab, 1963, nr. 62, 30.VI.1963; 1 ♂, Ostgobi aimag: 20 km SO of Čojren, 1200 m, Exp. Dr. Z. Kaszab, 1963, nr. 70, 1.VII.1963; 1 ♂, Sukhbaatar aimag: 44 km SSW of Baruun urt, 1050 m, Exp. Dr. Z. Kaszab, 1965, nr. 353, 3.VIII.1965; 1 ♀, Chentej aimag: 10 km W of Somon Delgerchaan, 1250 m, Exp. Dr. Z. Kaszab, 1965, nr. 476, 24.VIII.1965, allotype).
Philoctetes shokalskii : Kimsey and Bohart 1991: 257 (cat., Mongolia: Urga).

####### Material examined.

Mongolia: *Bayankhongor*, 5 ♀♀, 11 ♂♂, 86 km NW of Bayankhongor, 46°50'N, 100°04'E, 2070 m, 14.VII. 2004, leg. JS, MK, JH (MHC); *Dornod*, 1 ♀, 50 km SW of Choilbalsan, 960 m, 25.VII.2007, leg. JH (MHC); *Govi-Altai*, 1 ♀, 1 ♂, 70 km of Altay city, Guulin, 14.VII.2005, leg. JH (MHC); *Govi-Sümber*, 2 ♂♂, 20 km SE of Choyr, 1480 m, 7.VIII.2007, leg. MK (PRC); 1 ♀, 1 ♂, ibid, leg. MH (MHC); *Sukhbaatar*, 4 ♂♂, 200 km SSE of Baruun-Urt, Moltsoy Els, 1250 m, 27.VII.2007, leg. MK (PRC); 1 ♂, 210 km SSE of Baruun-Urt, 29.VII.2007, steppe, leg. MK (PRC); 12 ♂♂, 100 km SSW of Baruun-Urt, 1100 m, 30.VII.2007, leg. MH (MHC); *Tuv*, 1 ♀, Teregtin, 1350 m, Exp. Dr. Z. Kaszab, 1963, Nr.73, 2.VII.1963, det. Linsenmaier 1966 (NMLS); *Ulaanbaatar*, ♀ [not ♂], env. Urga [Ulaanbaatar], 1–4.VI.1909, leg. P. Kozlov, *Ellamp. shokalskii m*. [mihi] Typ. ♂. A. Semenov-Tian-Shansky det. V.19, Lectotype *Ellampus shokalskii* Sem. design. LS Kimsey <red label> (ZIN); 1 ♀, same data, paralectotype (ZIN); *Umnugovi*, 1 ♀, Gobi Gurvansaikhan National Park, 40 km W of Dalanzadgad, 2000 m, 28–30.VI. 2003, leg. JH (MHC); *Uvurkhangai*, 9 ♂♂, 12 km E of Aravaykheer, 46°22'N, 102°49'E, 1800 m, 3.VII.2004, leg. JH (MHC).

####### Distribution.

Mongolia (*Bayankhongor, *Dornod, Dornogovi, *Govi-Sümber, Khentii, Sukhbaatar,*Tuv, Ulaanbaatar, *Umnugovi, Uvurkhangai) (Kimsey 1986).

##### Genus *Pseudomalus* Ashmead, 1902

*Pseudomalus* Ashmead, 1902: 229. Type species: *Omalus semicircularis* Aaron, 1885 [= *Pseudomalus janus* (Haldeman, 1844)], by original designation.

###### Pseudomalus auratus nigridorsus

Taxon classificationAnimaliaHymenopteraChrysididae

(Tsuneki, 1953)

935EF421-D5F4-5670-81C1-D29CF280862A

Ellampus auratus f. nigridorsus Tsuneki, 1953a: 54. Syntypes ♂, ♀; Japan, Korea, Manchuria (NIAS).

####### Material examined.

Mongolia: *Bulgan*, 1 ♀, 137 km NE of Aravaykheer, 47°20'N, 103°45.5'E, 1250 m, 2.VII.2004, leg. JS (MHC); 1 ♀, Mongol Els Nat. Res., dunes, 47°24'N, 103°39'E, 31.VII.2005, leg. JH (MHC); *Khentii*, 1 ♀, 100 km NE of Ondorkhaan, Kerulen River, 970 m, 22.VII.2007, leg. MH (MHC); *Tuv*, 2 ♀♀, 18 ♂♂, 50 km E of Ulaanbaatar, Tuul River, 22.VI.2003, leg. JH (MHC); *Zavkhan*, 2 ♀♀, 40 km SW of Uliastay, dunes, 18.VII.2005, leg. JH (MHC).

####### Distribution.

*Mongolia (Bulgan, Khentii, Tuv, Zavkhan); Russia (Eastern Siberia, Far East), China, Korea, Japan (Rosa et al. 2019).

###### Pseudomalus corensis

Taxon classificationAnimaliaHymenopteraChrysididae

(Uchida, 1927)

6B40105F-768D-5161-A13A-2F46F6123712

Philoctetes punctatus var. corensis Uchida, 1927: 153. Holotype ♂; Korea: Seiryori (descr.) (NIAS).
Omalus joannisi du Buysson, 1908: Móczár 1967: 185. (cat., Mongolia: Central aimag: Zuun-Chara, 850 m, Exp. Dr. Z. Kaszab, 1964, nr. 281; 8.VII.1964) (mis.).

####### Material examined.

Mongolia: *Bulgan*, 1 ♀, Mongol Els Nat. Res., dunes, 47°24'N, 103°39'E, 31.VII.2005, leg. JH (MHC); *Dornod*, 1 ♀, 100 km W of Choilbalsan, 820 m, 23.VII.2007, leg. MH (MHC); 2 ♂♂, 20 km W of Choilbalsan, 800 m, 48°01'N, 114°14'E, 24.VII.2007, leg. MH (MHC/PRC); *Khentii*, 1 ♀, 1 ♂, 100 km NE of Ondorkhaan, Kerulen River, 970 m, 22.VII.2007, leg. MH (MHC); *Selenge*, 7 ♀♀, 5 ♂♂, 90 km N of Ulaanbaatar, Segnez River, 1450 m, 6–8.VII.2003, leg. JH (MHC/PRC); *Tuv*, 7 ♀♀, 3 ♂♂, 50 km N of Ulaanbaatar, E of Mandal, 1180 m, 8–13.VIII.2007, leg. MH (MHC, PRC); *Ulaanbaatar*, 3 ♀♀, Ulaanbaatar, Tuul River valley, 12.VII.2003, leg. JH (MHC).

####### Distribution.

Mongolia (*Bulgan, *Dornod, *Khentii, *Selenge, Tuv, *Ulaanbaatar); Russia (Eastern Siberia, Far East); Japan (Hokkaido) (Rosa et al. 2019).

####### Remarks.

The specimen illustrated in the volume of Russian cuckoo wasps (Rosa et al. 2019: fig. 18) is apparently misidentified and currently belonging to an unidentified species. Examination of type material by Uchida is needed for further studies.

###### Pseudomalus punctatus

Taxon classificationAnimaliaHymenopteraChrysididae

(Uchida, 1927)

3E3FC438-A781-5016-9809-7985AFE51E92

Philoctetes punctatus Uchida, 1927: 152. Syntypes ♂, ♀; Japan: Hokkaido and Honshu (NIAS?).
Omalus punctatus : Móczár, 1967: 185 (cat., Mongolia: 1 ♀, 1 ♂, Čojbalsan [= Dornod] aimag: Chamardavaa ul, 80 km SO of Somon Chalchingol, 600 m, Exp. Dr. Z. Kaszab, 1965, nr. 401, 13.VIII.1963).

####### Material examined.

Mongolia: *Bulgan*, 1 ♀, Mongol Els Nat. Res., dunes, 47°24'N, 103°39'E, 31.VII.2005, leg. JH (MHC); *Khentii*, 14 ♀♀, 100 km NE of Ondorkhaan, Kerulen River, 970 m, 22.VII.2007, leg. JH, MH (MHC); *Tuv*, 18 ♂♂, 50 km E of Ulaanbaatar, Tuul River, 22.VI.2003, leg. JH (MHC).

####### Distribution.

Mongolia (*Bulgan, Dornod, *Khentii, *Tuv); Russia (Eastern Siberia, Far East); Korea, Japan (Rosa et al. 2019).

###### Pseudomalus pusillus

Taxon classificationAnimaliaHymenopteraChrysididae

(Fabricius, 1804)

6260FC01-21A3-55BE-B156-45A6B3395518

Chrysis pusilla Fabricius, 1804: 176. Lectotype ♀ (designated by Rosa et al. 2020: 66); Austria (NHMW) (examined).
Omalus pusillus : Móczár, 1967: 195 (cat., Mongolia: 1 ♀, Čojbalsan [= Dornod] aimag: 50 km SO of Čojbalsan [= Choibalsan], 700 m, Exp. Dr. Z. Kaszab, 1965, nr. 421, 16.VIII.1965; 1 ♂, Čojbalsan [= Dornod] aimag: 44 km NW of Čojbalsan [= Choibalsan], 750 m, Exp. Dr. Z. Kaszab, 1965, nr. 425, 17.VIII.1965).

####### Material examined.

Mongolia: *Bayankhongor*, 2 ♀♀, 16 km SW of Bayankhongor, 46°13'N, 100°30'E, 2165 m, 10.VII.2004, leg. JS (MHC); *Bulgan*, 2 ♀♀, 137 km NE of Aravaykheer, 47°20'N, 103°45.5'E, 1250 m, 2.VII.2004, leg. JS (MHC); *Dornod*, 4 ♀♀, 5 ♂♂, 100 km W of Choilbalsan, 820 m, 23.VII.2007, leg. MH (MHC); 4 ♀♀, 20 km W of Choilbalsan, 800 m, 48°01'N, 114°14'E 24.VII.2007, leg. MH (MHC); 2 ♂♂, 50 km SW of Choilbalsan, 960 m, 25.VII.2007, leg. JH (MHC); *Khentii*, 3 ♀♀, 5 ♂♂, 100 km NE of Ondorkhaan, Kerulen River, 970 m, 22.VII.2007, leg. MH (MHC); *Selenge*, 2 ♂♂, 90 km N of Ulaanbaatar, Segnez River, 1450 m, 6–8.VII.2003, leg. JH (MHC); *Tuv*, 2 ♂♂, 50 km E of Ulaanbaatar, Tuul River, 22.VI.2003, leg. JH (MHC); *Umnugovi*, 3 ♀♀, 12 ♂♂, Gobi Gurvansaikhan National Park, 40 km W of Dalanzadgad, 2000 m, 28–30.VI.2003, leg. JH (MHC); *Uvurkhangai*, 4 ♀♀, 13 ♂♂, 12 km E of Aravaykheer, 46°22'N, 102°49'E, 1800 m, 3.VII.2004, leg. JH (MHC).

####### Distribution.

Mongolia (*Bayankhongor, *Bulgan, Dornod, *Khentii, *Selenge, *Tuv, *Umnugovi, *Uvurkhangai); Palaearctic, from western Europe and northern Africa to Russian Far East (Kurzenko and Lelej 2007).

#### Tribe Parnopini

##### Genus *Parnopes* Latreille, 1797

*Parnopes* Latreille, 1797: 126. Type species: *Chrysis carnea* Fabricius, 1775 [= *Parnopes grandior* (Pallas, 1771)], by monotypy.

###### Parnopes glasunowi

Taxon classificationAnimaliaHymenopteraChrysididae

Semenov, 1901

F1D126B9-5C46-5E88-8C82-1E798FC161C3

Parnopes glasunowi Semenow, 1901: 25. Holotype ♂; Tajikistan: “Turkestan occid.[entalis]: Jagnob: Rovat, 12.VII.1892, leg. D. Glasunow” (ZIN) (examined).

####### Material examined.

Mongolia: *Khovd*, 1 ♂, ur. Elkhon, 20 km SE of Altai, Bodoncha, 27.V.1970, leg. E. Narchuk (ZIN).

####### Distribution.

*Mongolia (Khovd); Central Asia, Russia (south of European part) (Rosa et al. 2019).

###### Parnopes popovii

Taxon classificationAnimaliaHymenopteraChrysididae

Eversmann, 1858

5F9B12C8-F505-5FB1-A538-5E512D7429EC

Parnopes popovii Eversmann, 1858: 567. Holotype ♀; Russia: Siberia “campis orientalibus” (ISEA-PAS) (examined). Kimsey and Bohart 1991: 586 (cat., Mongolia, without locality).

####### Material examined.

Mongolia: *Arkhangai*, 1 ♂, 25 km NE of Tsetserleg, 47°38'N, 101°45'E, 23.VII.2004, leg. JH (MHC); 1 ♂, 90 km NE of Tsetserleg, 48°03'N, 102°25'E, 24.VII.2004, leg. JH (MHC); 1 ♀, ibid, 27.VII.2005, leg. JH (MHC); *Bulgan*, 1 ♂, 143 km NE of Arvaykheer, 47°24'N, 103°39'E, 26.VII.2004, 1300 m, sandy dunes, JS (PRC); 4 ♀♀, 3 ♂♂, Mongol Els Nat. Res., 47°24'N, 103°39'E, dunes, 1320 m, 31.VII.2005, leg. JH (PRC); *Dornod*, 2 ♂♂, 50 km SW of Choibalsan, 960 m, 25.VII.2007, leg. JH (MHC); *Dornogovi*, 1 ♀, 2 ♂♂, 28 km SE of Chatan-Bulag, 3.VIII.2007, leg. MH (MHC); *Sukhbaatar*, 4 ♀♀, 4 ♂♂, 200 km SSE of Baruun-Urt, Moltsoy Els, 1250 m, 27.VII.2007, leg. MH (MHC); 1 ♂, 3 ♀♀, ibid, 27.VII.2007, leg. JH (MHC); 2 ♂♂, 100 km SSW of Baruun-Urt, 1100 m, 30.VII.2007, leg. MH (MHC); *Tuv*, 1 ♀, 75 km W of Ulaanbaatar, dunes, 2.VIII.2005, leg. JH (MHC); *Umnugovi*, 1 ♀, Gobi, Dalanzadgad, 25.VI.2003, leg. JH (MHC).

####### Distribution.

Mongolia (*Arkhangai, *Bulgan, *Dornod, *Dornogovi, *Sukhbaatar, *Tuv, *Umnugovi); China (Heilongjiang, Shanghai, Shandong), Korea, Russia (Rosa et al. 2014).

### Species to be excluded from Mongolian fauna

The following 19 taxa were described or listed for Mongolia by Radoszkowski (1887, 1891), Mocsáry (1890), Dalla Torre (1892), du Buysson (1893), Bischoff (1913), Linsenmaier (1959), and Kimsey and Bohart (1991), yet the type localities are situated in Inner Mongolia (China) or Central Asian countries. These species are expected in Mongolia due to the close vicinity of the collecting localities, with the only exception of *Chrysis fouqueti* (du Buysson, 1909), which belongs to the Oriental fauna.

***Elampus mocsaryi* Radoszkowski, 1887**

*Elampus mocsari* (!) Radoszkowski, 1887: 45. Holotype ♀; Mongolia [= China]: Qinghai: Zaïdam (ISEA-PAS) (examined).

Ellampus (Notozus) mocsaryi: Mocsáry 1889: 80. Justified emendation of *Elampus mocsari* Radoszkowski, 1887. Dalla Torre 1892: 14 (cat., Mongolia [= China]).

*Ellampus mocsaryi*: Dalla Torre 1892: 14 (cat., Mongolia [= China]); Kimsey and Bohart 1991: 168 (cat., Mongolia [= China]: Zaidam).

*Notozus mocsaryi*: Bischoff 1913: 6 (cat., Mongolia [= China]).

Omalus (Notozus) mocsaryi: Linsenmaier 1959: 16 (key), 24 (tax., descr., Mongolia [= China]).

***Elampus spinipes* (Mocsáry, 1890)**

Ellampus (Notozus) spinipes Mocsáry, 1890: 49. Holotype ♀; Mongolia [= China, Inner Mongolia]: Mongolia meridionalis (Ta-Wan) (ISEA-PAS) (examined).

*Ellampus spinipes*: Dalla Torre 1892: 18 (cat., Mongolia [= Inner Mongolia]).

*Elampus spinipes*: Kimsey and Bohart 1991: 171 (cat., Mongolia [= Inner Mongolia]: Ta-Wan).

*Notozus spinipes*: Bischoff 1913: 7 (cat., Mongolia [= Inner Mongolia]).

Omalus (Notozus) spinipes: Linsenmaier 1959: 16 (key), 24 (descr., Mongolia [= Inner Mongolia]).

***Hedychridium ardens mongolicum* Tsuneki, 1947**

Hedychridium ardens f. mongolicum Tsuneki, 1947: 47. Holotype ♀; China: Inner Mongolia: Apaka (NIAS).

Hedychridium ardens f. mongolicum: Kimsey and Bohart 1991: 188 (cat., Mongolia [= China, Inner Mongolia]: Apaka).

***Hedychrum simile* Mocsáry, 1889**

*Hedychrum cyaneum* Mocsáry in Radoszkowski 1889: 10, nec Brullé, 1846. Lectotype ♀ (designated by French, in Bohart and French 1986: 341); China “Ta-schian-sy” (HNHM) (examined).

*Hedychrum simile* Mocsáry, 1889: 157. Replacement name for *Hedychrum cyaneum* Radoszkowski, 1889, nec Brullé, 1846.

Hedychrum simile f. mongolicus Tsuneki, 1947: 54. Syntypes ♀♀, ♂♂; China: Inner Mongolia: Apaka (NIAS).

*Hedychrum simile*: Linsenmaier 1959: 39 (descr., key, Mongolia [= China, Inner Mongolia]); Kimsey and Bohart 1991: 220. Mongolia (cat., without locality, related to the record of *H. simile mongolicus* from Inner Mongolia by Tsuneki 1947).

***Philoctetes hypocrita* (du Buysson, 1893)**

*Ellampus hypocrita* du Buysson, 1893: 246. Syntypes ♀♀; Mongolia [= China, Inner Mongolia], Kansu-Jelisyn-Kuse (ISEA-PAS); Persia (MNHN) (examined). Bischoff 1913: 8 (cat., Mongolia [= China, Inner Mongolia])

*Omalus hypocritus*: Kimsey and Bohart 1991: 248. Incorrect subsequent spelling.

*Pseudomalus hypocrita*: Rosa et al. 2015: 77.

*Philoctetes hypocrita*: Farhad et al. 2018: 199. Lectotype designation: ♂; China: Kansu Jelisyn Kuse (ISEA-PAS).

***Pseudomalus tshingiz* (Semenov, 1954)**

*Ellampus tshingiz* Semenov in Semenov-Tian-Shanskji and Nikol’skaya, 1954: 93. Holotype ♂; China: Sandzhu [Xinjiang], Gushan Gobi (depository: ZIN) (examined). Rosa et al. 2017a: 80 (cat., type series), 221 (plate 223).

*Pseudomalus tshingiz*: Kimsey and Bohart 1991: 270 (cat., Mongolia [= China]: Sachow Gobi [= Oasis Sachzhou, Gashunskoe Gobi [= Dunhuang, Gansu]).

***Chrysis aegle* Semenov, 1967**

Chrysis (Gonodontochrysis) aegle Semenov-Tian-Shanskij, 1967: 160. Holotype ♀; China: Alashan, Maladzhin (ZIN) (examined).

*Chrysis aegle*: Kimsey and Bohart 1991: 379 (cat., Mongolia [= China, Inner Mongolia]: Alashan, Maladzhin).

***Chrysis analis altaica* Mocsáry, 1912**

Chrysis (Tetrachrysis) analis var. altaica Mocsáry, 1912: 586. Holotype ♀; Kazakhstan: Altai: Semipalatinsk (HNHM) (examined).

*Chrysis altaica*: Kimsey and Bohart 1991: 381 (cat., Mongolia [= Kazakhstan]: Altai Mts).

***Chrysis fouqueti* (du Buysson, 1909)**

*Tetrachrysis fouqueti* du Buysson, 1909: 210. Holotype ♀; Viet Nam: Tonkin (MNHN).

*Chrysis fouqueti*: Kimsey and Bohart 1991: 412 (cat., Mongolia for the erroneous synonymy of *Chrysis csikiana* Mocsáry, 1912).

***Chrysis csikiana* Mocsáry, 1912**

*Chrysis (Tetrachrysis) Csikiana* Mocsáry, 1912: 406. Lectotype ♂ (designated by Bohart in Bohart and French 1986: 341); Kazakhstan: Semipalatinsk (HMNH) (examined).

*Chrysis csikiana*: Kimsey and Bohart 1991: 412 (cat., Mongolia [= Kazakhstan]: Altai Mts).

***Chrysis jelisyni* Radoszkowski, 1891**

*Chrysis jelisyni* Radoszkowski, 1891: 186. Syntypes ♀♀; Mongolia [= China]: Kansu, Jelissyn-Kuce (ISEA-PAS, MfN) (examined).

Chrysis (Tetrachrysis) jelisyni: Bischoff 1913: 54 (cat., Mongolia [= China]: Totau (locality not found)).

*Chrysis jelisyni*: Kimsey and Bohart 1991: 34 (cat., Mongolia [= China]: Kansu).

***Chrysis keriensis* Radoszkowski, 1887**

Chrysis (Tetrachrysis) keriensis Radoszkowski, 1887: 47. Holotype ♂ [not ♀]; China: Xinjiang, Keria-Daria (ISEA-PAS) (examined).

Chrysis (Tetrachrysis) keriensis: Mocsáry 1889: 516 (tax., descr., Mongolia [= China]); Bischoff 1913: 54 (cat., Mongolia [= China]).

*Chrysis keriensis*: Dalla Torre 1892: 73 (cat., Mongolia [= China]); Kimsey and Bohart 1991: 427 (cat., Mongolia [= China]: Keria Daria).

***Chrysis kozlovi* Semenov, 1967**

Chrysis (Gonodontochrysis) kozlovi Semenov-Tian-Shanskij, 1967: 160. Holotype ♂; China: Alashan, Uzosto canyon, 14.5.1908, leg. P. Kozlov (ZIN) (examined).

*Chrysis kozlovi*: Kimsey and Bohart 1991: 429 (cat., Mongolia [= China, Inner Mongolia]: Alashan, Uzosto Canyon).

***Chrysis mongoliana* Bohart, 1991**

Chrysis (Tetrachrysis) mongolica Semenov-Tian-Shanskij, 1967: 178, nec Mocsáry, 1914. Holotype ♀; Russia: Transbaikalia: Ingoda River (ZIN) (examined).

*Chrysis mongoliana* Bohart in Kimsey and Bohart 1991: 440. Replacement name for Chrysis (Tetrachrysis) mongolica Semenov-Tian-Shanskij, 1967, nec Mocsáry, 1914 (cat., Mongolia: Transbaikalia: Ingoda river).

***Chrysis potanini* Radoszkowski, 1891**

*Chrysis potanini* Radoszkowski, 1891: 186. Holotype ♂; Mongolia [= China]: Tufyn (ISEA-PAS) (examined).

Chrysis (Tetrachrysis) potanini: Bischoff 1913: 57 (cat., Mongolia [= China]).

*Chrysis potanini*: Kimsey and Bohart 1991: 450 (cat., Mongolia [= China]: Tufyn).

***Chrysis przewalskii* Radoszkowski, 1887**

*Chrysis Przewalskii* Radoszkowski, 1887: 46. Holotype ♂; Mongolia [= China]: Zaïdam (ISEA-PAS) (examined).

Chrysis (Tetrachrysis) przewalskii: Mocsáry 1889: 504 (tax., descr., Mongolia [= China]); Bischoff 1913: 57 (cat., Mongolia [= China]).

*Chrysis przewalskii*: Dalla Torre 1892: 86 (cat., Mongolia [= China]); Kimsey and Bohart 1991: 452 (cat., Mongolia [= China]: Zaidam, Keria Mts).

***Chrysis spinidens* Mocsáry, 1887**

Chrysis (Tetrachrysis) spinidens Mocsáry in Radoszkowski, 1887: 48. Holotype ♂; Mongolia [= China]: Zaïdam (ISEA-PAS) (examined). Mocsáry 1889: 516 (cat., descr., Mongolia [= China]); Bischoff 1913: 59 (cat., Mongolia [= China]).

*Chrysis spinidens*: Dalla Torre 1892: 97 (cat., Mongolia [= China]); Kimsey and Bohart 1991: 464 (cat., Mongolia: Zaidam).

***Chrysura alticola* (Semenov-Tian-Shanskij, 1912)**

*Chrysis petri alticola* Semenov-Tian-Shanskij, 1912: 190. Lectotype ♀ (designated by Rosa et al. 2017a: 45); Kyrgyzstan: Peter the Great Range, Gardan-Kaftar Pass (ZIN) (examined).

*Chrysura alticola*: Kimsey and Bohart 1991: 486 (cat., Mongolia [= Kyrgyzstan]).

## Conclusions

Approximately 1500 chrysidid specimens were examined for the compilation of this first checklist of the Mongolian Chrysididae. Fifty-seven resulted newly recorded, but still a large number of specimens are laying unidentified in museum and private collections. Nineteen species were excluded from the fauna of Mongolia, because collecting localities are currently included in China territories; however, these species are expected for Mongolia. Based on the available data, distributional records for 90 Mongolian species are listed, representing 18 genera grouped in two subfamilies. In terms of species richness, Cleptinae are represented only by two species so far, and the subfamily Chrysidinae is the most speciose (88 species, 98%). Among Chrysidinae, Chrysidini is the most speciose tribe (47 species, 53.4%), followed by Elampini (39 species, 44.3%), and finally Parnopini (2 species, 2.2%).

Currently eight species (9% of known taxa) are provisionally considered endemic: *Cleptes mongolicus* Rosa, Halada & Agnoli, sp. nov., *Chrysis mocsaryi* Radoszkowski, 1889, *Ch. priapus* Rosa, 2018, *Spinolia spinosa* Rosa & Halada, sp. nov., *Elampus spinifemoris* (Móczár, 1967), *Hedychrum lama* du Buysson, 1891, *Holopyga kaszabi* Móczár, 1967, and *Philoctetes shokalskii* (Semenov, 1932).

From a chorological point of view, one species has a Holarctic distribution (*Pseudochrysis neglecta*), ten have Palaearctic distributions, one has a Holarctic and Oriental distribution (*Omalus aeneus*), one a Palaearctic and Oriental distribution (*Hedychridium gerstaeckeri*), 28 species have an Asiatic-European distribution, 21 have an East Palaearctic distribution, and 19 have a Central Asian distribution.

Another result of the present study is a better knowledge of the distributional limits of some species, and Mongolia represents the easternmost record for seven species: *Chrysis aestiva*, *Ch. illigeri*, *Ch. jaxartis*, *Ch. leptomandibularis*, *Chrysura ignifrons*, *Elampus albipennis* and *Philoctetes bogdanovii*.

The most widespread Mongolian species is the endemic *Philoctetes shokalskii* recorded in ten aimags. *Chrysis consanguinea* and *Hedychridium ardens* were recorded in nine aimags. This is not surprising because *C. consanguinea* resulted in being one of the most common species from Western to Eastern Siberia also (Rosa et al. 2017c, d), whereas *H. ardens* is one of the most common Euro-Siberian species ranging from Central Europe to China (Rosa et al. 2014). *Pseudomalus pusillus* was recorded in eight aimags, whereas *H. cupreum*, *H. chalybaeum* and *Philoctetes mongolicus* were recorded in seven7 aimags; they are Asiatic-European species, sometimes locally abundant. *Hedychridium longigena* and *Parnopes popovii* were recorded in seven aimags, yet they are East-Palaearctic species.

Although most of the Mongolian aimags are under-represented in the existing data due to inadequacy of surveys, based on the currently available data we can state that the highest number of recorded species was collected in Tuv (42 species), Arkhangai (27 species), and Selenge and Dornod (20 species) aimags (Table 2). The family Chrysididae has not yet been documented from Bayan-Ulgii, Darkhan-Uul, Dundgovi, Khuvsgul, Orkhon, and Uvs although it is probable that this cosmopolitan family is present in these aimags and it is only a matter of time before the fauna is sampled and recorded.

**Table 2.** Species diversity of aimags in terms of area size, number of specimens and number of collecting sites.

Aimags	Area, km^2^	No. of species	No. of coll. sites	No. of specimens
Arkhangai	55 314	27	4	237
Bayankhongor	115 978	18	10	142
Bulgan	48 733	18	4	61
Dornod	123 597	19	4	228
Dornogovi	109 472	16	4	42
Govi-Altai	141 448	8	5	49
Govi-Sümber	5 542	1	1	4
Khentii	80 325	12	1	55
Khovd	76 061	4	4	4
Selenge	41 153	20	1	100
Sukhbaatar	82 286	17	4	79
Tuv	74 042	42	10	350
Ulaanbaatar	4 704	10	4	20
Umnugovi	165 381	13	7	34
Uvurkhangai	62 895	12	5	42
Zavkhan	82 457	11	1	21

Comment. Chrysididae are not known in Bayan-Ulgii, Darkhan-Uul, Dundgovi, Khuvsgul, Orkhon, and Uvs.

Overall, the Mongolian fauna is still too poorly known for a complete analysis of species richness and composition. The faunal richness of Mongolia is doubtless much higher than we currently know, in comparison with the chrysidid fauna of the adjacent countries and considering the geographic position of Mongolian aimags and their different biotopes. For example, at least another 75 species recorded for Siberia (Rosa et al. 2017d) are expected for Mongolia, as well as other five genera known from bordering and central Asian countries (Chrysidini: *Chrysidea* Bischoff, 1913, *Spintharina* Semenov, 1892; Elampini: *Chrysellampus* Semenov, 1932, *Haba* Semenov, 1954; Parnopini: *Cephaloparnops* Bischoff, 1910). Copious undescribed members of the genus *Prochridium* Linsenmaier, 1968 were collected in Mongolia. The descriptions of Mongolian *Prochridium*, and a revision of this genus, are in preparation. Only a single record of an undescribed *Prochridium* was previously known in literature for Central Asia (Turkmenistan) (Linsenmaier 1994).

## Supplementary Material

XML Treatment for Cleptes dauriensis

XML Treatment for Cleptes mongolicus

XML Treatment for Chrysis aestiva

XML Treatment for Chrysis angustula

XML Treatment for Chrysis asahinai

XML Treatment for Chrysis belokobylskiji

XML Treatment for Chrysis brevitarsis

XML Treatment for Chrysis castigata

XML Treatment for Chrysis chinensis

XML Treatment for Chrysis consanguinea

XML Treatment for Chrysis dauriana

XML Treatment for Chrysis equestris

XML Treatment for Chrysis fulgida

XML Treatment for Chrysis ignita

XML Treatment for Chrysis illecebrosa

XML Treatment for Chrysis illigeri

XML Treatment for Chrysis ismaeli

XML Treatment for Chrysis jaxartis

XML Treatment for Chrysis leptomandibularis

XML Treatment for Chrysis mane

XML Treatment for Chrysis matutina

XML Treatment for Chrysis mediata

XML Treatment for Chrysis mocsaryi

XML Treatment for Chrysis mysticalis

XML Treatment for Chrysis nox

XML Treatment for Chrysis pavesii

XML Treatment for Chrysis priapus

XML Treatment for Chrysis pseudobrevitarsis

XML Treatment for Chrysis pupilla

XML Treatment for Chrysis rutilans

XML Treatment for Chrysis schencki

XML Treatment for Chrysis sibirica

XML Treatment for Chrysis solida

XML Treatment for Chrysis splendidula unica

XML Treatment for Chrysis subcoriacea

XML Treatment for Chrysis viridula

XML Treatment for Chrysura dichroa

XML Treatment for Chrysura ignifrons

XML Treatment for Euchroeus mongolicus

XML Treatment for Euchroeus orientis

XML Treatment for Pentachrysis amoena

XML Treatment for Pseudochrysis gengiskhan

XML Treatment for Pseudochrysis neglecta

XML Treatment for Spinolia spinosa

XML Treatment for Spinolia unicolor

XML Treatment for Stilbum calens

XML Treatment for Trichrysis cyanea

XML Treatment for Trichrysis pellucida

XML Treatment for Trichrysis secernenda

XML Treatment for Colpopyga nesterovi

XML Treatment for Elampus albipennis

XML Treatment for Elampus coloratus

XML Treatment for Elampus montanus

XML Treatment for Elampus panzeri

XML Treatment for Elampus sanzii

XML Treatment for Elampus spinifemoris

XML Treatment for Hedychridium ardens

XML Treatment for Hedychridium asianum

XML Treatment for Hedychridium belokobylskiji

XML Treatment for Hedychridium cupreum

XML Treatment for Hedychridium gabriellae

XML Treatment for Hedychridium longigena

XML Treatment for Hedychridium propodeale

XML Treatment for Hedychridium roseum

XML Treatment for Hedychrum chalybaeum

XML Treatment for Hedychrum gerstaeckeri

XML Treatment for Hedychrum lama

XML Treatment for Hedychrum longicolle

XML Treatment for Hedychrum nobile

XML Treatment for Hedychrum rutilans ermak

XML Treatment for Holopyga generosa asiatica

XML Treatment for Holopyga kaszabi

XML Treatment for Holopyga minuma

XML Treatment for Omalus aeneus

XML Treatment for Omalus berezovskii

XML Treatment for Omalus margianus

XML Treatment for Omalus miramae

XML Treatment for Omalus stella

XML Treatment for Philoctetes bogdanovii

XML Treatment for Philoctetes cynthiae

XML Treatment for Philoctetes diakonovi

XML Treatment for Philoctetes lyubae

XML Treatment for Philoctetes mongolicus

XML Treatment for Philoctetes shokalskii

XML Treatment for Pseudomalus auratus nigridorsus

XML Treatment for Pseudomalus corensis

XML Treatment for Pseudomalus punctatus

XML Treatment for Pseudomalus pusillus

XML Treatment for Parnopes glasunowi

XML Treatment for Parnopes popovii
